# EphA2-targeted alpha-particle theranostics for enhancing PDAC treatment

**DOI:** 10.7150/thno.106948

**Published:** 2025-03-18

**Authors:** Ajay Kumar Sharma, Kuldeep Gupta, Akhilesh Mishra, Gabriela Lofland, Sophia Y. Chen, Ian Marsh, Peyton T Fair, Robert F. Hobbs, Todd M. Armstrong, Elizabeth M. Jaffee, Edward W. Gabrielson, Lei Zheng, Sridhar Nimmagadda

**Affiliations:** 1 The Russell H. Morgan Department of Radiology and Radiological Science, Johns Hopkins University School of Medicine, Baltimore, MD, 21287, USA.; 2 Department of Oncology, The Sidney Kimmel Comprehensive Cancer Center and the Bloomberg-Kimmel Institute for Cancer Immunotherapy, Johns Hopkins University School of Medicine, Baltimore, MD, 21287, USA.; 3 Department of Pathology and Oncology, Johns Hopkins University School of Medicine, Baltimore, MD, 21287, USA.; 4 Department of Pharmacology and Molecular Sciences, Johns Hopkins University School of Medicine, Baltimore, MD, 21287, USA.; 5 Division of Clinical Pharmacology, Department of Medicine, Johns Hopkins University School of Medicine, Baltimore, MD, 21287, USA.

**Keywords:** Pancreatic cancer, PET, Imaging, Radiotherapy, Peptide radiopharmaceuticals, Gallium-68, Alpha-particle therapy

## Abstract

**Background:** Pancreatic ductal adenocarcinoma (PDAC) presents a formidable challenge in oncology due to its aggressive nature and resistance to therapy. Current treatments, including surgery, chemotherapy, and radiotherapy, have limited success in improving patient outcomes. This study addresses the urgent need for novel radiotheranostic strategies for PDAC by investigating EphA2 as a potential target.

**Methods and Results:** Analysis of genomic data from the Cancer Cell Line Encyclopedia (CCLE) and The Cancer Genome Atlas (TCGA) revealed elevated EphA2 expression in PDAC, confirmed by immunohistochemical staining of tumor tissue microarrays (TMAs). Further analysis showed variable EphA2 expression across PDAC cell lines, with surface receptor density not always correlating with mRNA levels. A low molecular weight peptide was developed and labeled with gallium-68 for PET imaging. *In vitro* studies demonstrated specific binding to EphA2-expressing PDAC cells with rapid internalization. *In vivo* PET imaging in subcutaneous and orthotopic PDAC models confirmed high tumor uptake and minimal off-target binding, confirming EphA2 as a valid imaging target. For molecular radiotherapy, a DOTA-conjugated peptide was labeled with the alpha-particle emitter, actinium-225. *In vitro* studies revealed dose-dependent cytotoxicity in PDAC cells, with an IC_50_ of 0.32 µCi/mL. In a tumor model, treatment with Ac-225 labeled peptide significantly inhibited tumor growth compared to controls, with mild adverse effects.

**Conclusion:** These results establish EphA2 as a promising radiotheranostic target in PDAC, with potential for both non-invasive imaging and targeted radiotherapy. Given the potential, further optimization of EphA2-targeted agents are warranted to advance personalized treatment strategies for PDAC patients.

## Introduction

Pancreatic ductal adenocarcinoma (PDAC) is a recalcitrant disease with a mortality rate nearly equivalent to its incidence rate 1, 2. Most PDAC patients present with distant metastasis at diagnosis and only about 12% are predicted to survive beyond 5 years 1. This dismal outlook stems from the disease's elusive nature, marked by an inability to detect it early coupled with high therapeutic resistance attributed to the dense desmoplastic stroma and immune suppressive microenvironment characteristic of PDAC 3.

Current clinical management of PDAC involves a triad of approaches including surgery, systemic chemotherapy, and radiotherapy 1. However, while these modalities have shown efficacy in certain contexts, they often fall short in significantly improving patient outcomes. Despite the groundbreaking successes witnessed with checkpoint immunotherapies and molecular radiotherapy in other solid malignancies, their translation to PDAC has been met with limited success 3, 4. The promise of molecular radiotherapy, which is transforming the landscape of advanced cancer management, particularly evident in prostate and neuroendocrine cancers 5, remains largely unfulfilled in PDAC due to the scarcity of suitable radiotheranostic targets and agents tailored to this aggressive disease.

Radiopharmaceutical therapy (RPT) has emerged as a promising treatment approach for multifocal disease, delivering targeted radiation to cancer cells across disseminated disease sites while sparing healthy tissues 6, 7. Among RPT strategies, targeted alpha-emitter therapy (TAT) stands out, as it induces largely irreparable double-strand DNA breaks, leading to highly selective cytotoxicity in cancer cells 8, 9. Actinium-225 (^225^Ac) is a potent alpha emitting radionuclide used in TAT due to its high linear energy transfer (LET), leading to potent cancer cell killing, and its short path length (around 100 µm), minimizing off-target toxicity 10, 11. Preliminary data suggest that alpha-emitting radionuclides have a significantly lower impact on kidney function compared to beta-emitting radionuclides 12, although, additional studies are required to assess long-term renal toxicity. The clinical use of ^225^Ac could also be impacted by restricted availability, high production costs, and the complex management of its radioactive decay products, which can cause unintended radiation exposure. Despite these challenges, ^225^Ac-based therapies, such as [^225^Ac]Ac-PSMA-617 for metastatic prostate cancer and [^225^Ac]Ac-DOTATATE for neuroendocrine tumors, are demonstrating significant promise, especially for patients resistant to beta-emitting therapies or with radioresistant tumors 13, 14. These approaches, which are in various stages of clinical and preclinical evaluation, underscore the potential for developing similar therapeutic options for PDAC.

Given these advancements, there is a pressing need for the development of novel radiotheranostic approaches specifically tailored to address the challenges of PDAC. Low molecular weight peptide-based imaging agents, such as those targeting somatostatin receptor 2 (SSTR2) and fibroblast activation protein (FAP), have shown some utility in PDAC 6, 7. Nonetheless, the repertoire of such agents remains limited for PDAC, particularly in the realm of effective radiotherapeutics. One promising target is EphA2 (Ephrin receptor A2), which is highly expressed in PDAC and implicated in cancer progression through its roles in cell adhesion, migration, proliferation, and the maintenance of the tumor's immune suppressive microenvironment 15. Although antibody-based agents have been developed for EphA2 16, low molecular weight agents offer better pharmacokinetics and improved tumor penetration, opening new avenues for targeted molecular radiotherapy in PDAC 17, 18.

Previous efforts to target EphA2 have focused on developing antibody constructs for imaging. Examples include [^64^Cu]DOTA-1C1 mAb, which binds both human and mouse EphA2 and has been used for noninvasive PET imaging of EphA2 in colorectal, melanoma, glioblastoma, and ovarian cancers 19. Radiolabeled analogs of other anti-EphA2 antibodies, such as DS-8895a and 4B3, have similarly shown promise in detecting EphA2 expression non-invasively 20, 21. Despite these successes, antibody-based agents have limitations, such as suboptimal pharmacokinetics and high production costs. Low molecular weight agents exhibit tractable pharmacokinetics, improved tumor penetration, reduced immunogenicity, and lower production costs. Peptide-based agents like [^18^F]AFP-SWL, [^99m^Tc]-HYNIC-SWL, and [^68^Ga]DOTA-SD01 have shown specificity for EphA2 in melanoma, non-small cell lung, and breast cancers, but issues with plasma degradation and low tumor uptake have limited their use 22-24. Recently developed high-affinity bicyclic peptides, including [^18^F]AlF-ETN and [^68^Ga]Ga-BCY18469, demonstrate *in vivo* stability and enhanced imaging contrast in prostate and fibrosarcoma models 25, 26, further highlighting the potential of EphA2 as a radiotheranostic target.

In this study, we report the validation of EphA2 as a radiotheranostic target in PDAC. Leveraging genomic data sets and immunohistochemical (IHC) analyses of human tumor samples, we demonstrate the potential of EphA2 as a molecular radiotherapy target in PDAC. We detail the development of gallium-68 ([^68^Ga]) labeled peptide-based radiotracers for PET imaging, evaluating their pharmacokinetics, biodistribution, and EphA2 specificity both *in vitro* and *in vivo* across a spectrum of PDAC models. Additionally, we introduce an Actinium-225 ([^225^Ac]) radiotherapeutic analog and assess its efficacy in controlling tumor growth in PDAC models, thereby paving the way for targeted therapeutic interventions in this recalcitrant disease.

## Methods

### CCLE and human genomic database mining for EphA2 expression

Data was collected from CCLE (cancer cell line encyclopedia on 11-05-2021: https://depmap.org/portal/ccle/) for EphA2 mRNA expression. These data points were sorted for each cancer types, 14 cohorts were selected and rearranged in the descending order of median EphA2 mRNA expression. Human genomic database (TCGA) was collected using UCSC Xena interface (collected on 05-19-2021 from https://xenabrowser.net/) for expression of EphA2 mRNA in 34 cancer types and 18 cohorts were chosen for analysis. Median of these 18 cohorts was calculated and arranged in descending order. Similarly, to compare expression of EphA2 gene in healthy and primary tumors, 15 cohorts were chosen and arranged in descending order. All data were plotted in GraphPad prism 9.0.

### EphA2 immunohistochemistry of TMA233

For immunohistochemistry, tissue slides were heated to 60 °C and washed with xylene and alcohol to remove paraffin. Antigen retrieval was carried out using citrate buffer (pH 6.0, 95-100 °C, 20 min) and the endogenous peroxidase and alkaline phosphatase activity was blocked using BioXALL. The primary anti-human EphA2 antibody was applied at a dilution of 1:250 and incubated overnight at 4 °C (Cell Signaling Cat# 6997S). After washing slides with PBS, the secondary antibody, Signalstain Boost IHC Detection Reagent (HRP), was applied and incubated for 30 min at room temperature. The slides were then washed again with PBS and the stain was developed using ImmPACT DAB substrate (Vector Lab #SK4105) according to manufacturer's instructions, counterstained with Mayer's Hematoxylin for 1 min, dehydrated using alcohol and xylene, and cover slipped. The scoring of EphA2 was determined by the intensity and distribution of staining in tumor cells. A score of 0 indicates no staining or weak staining in less than 10% of tumor cells, while a score of 1 reflects weak to moderate membranous or membranous and cytoplasmic staining in more than 10% of tumor cells. A score of 2 signifies moderate membranous or membranous and cytoplasmic staining in more than 50% of tumor cells, and a score of 3 represents strong membranous or membranous and cytoplasmic staining in more than 80% of tumor cells.

### Chemicals

AJ200 and AJ201 were synthesized by CPC Scientific Inc. (Sunnyvale, CA) with >90% purity and further characterized by MALDI-TOF-MS. NOTA-NHS and DOTA-NHS esters were purchased from CheMatech and Macrocyclics, respectively.

### Synthesis of AJ201

AJ201 is a bicyclic peptide, contains 24 amino acid chains and is conjugated with a 2,2′,2”-(1,4,7-triazacyclononane-1,4,7-triyl)triacetic acid (NOTA) as a bifunctional chelator. The sequence of AJ201 peptide is NOTA-PEG_2_-GR-[SAR_3_]-GA-[hArg]-DC-[HyP]-LVNPLCLHP-[dD]-W-[hArg]-C-NH_2_ with TATA-based cyclization. AJ201 was characterized by MALDI-TOF-MS (**Figure S2**). Theoretical chemical formula: C_143_H_227_N_45_O_41_S_3_; Exact mass: 3326.62; Molecular weight: 3328.84; and the observed MALDI-TOF-MS mass [M + H]^+^ is 3329.0. This peptide sequence was derived from reported peptide 17, 18.

### Synthesis of AJ210

To a stirred solution of AJ200 (3.2 mg, 1.2 µmol) in 400 µL of DMF in a reaction vial, DOTA-NHS ester (2.0 mg, 2.6 µmol) and DIPEA (5 µL, 25 µmol) were added and stirred at room temperature for 3 h (**Scheme S1**). DMF was evaporated using a rotary evaporator under reduced pressure and the residual product was purified on a reversed phase high performance liquid chromatography, (RP-HPLC) system using a semi-preparative C-18 Luna column (5 mm, 10 x 250 mm Phenomenex, Torrance, CA). The HPLC condition was gradient elution started with 5% acetonitrile: water (0.1% TFA) and reached at 95% acetonitrile: water (0.1% TFA) in 20 min at a flow rate of 5 mL/min. The product AJ210 was collected at RT ~10.3 min, lyophilized to form an off-white powder (72% yield) and characterized by MALDI-TOF-MS (**Figure S10**). Theoretical chemical formula: C_131_H_203_N_41_O_39_S_3_; Exact mass: 3070.43; Molecular weight 3072.49; and the observed MALDI-TOF-MS mass [M + H]^+^ is 3072.6.

### *In vitro* binding affinity study of AJ201 and AJ210 with purified hEphA2 and mEphA2 by SPR

All experiments were conducted using a Biacore T200 instrument (GE Healthcare Life Sciences) with a CM5 chip at 25 °C. His-tagged human EphA2 (R&D systems, catalog # 3035-A2, 56.9 kDa, 4.46 μM stock concentration) and mouse EphA2 (SinoBiological, cat.# 50586-M08H, 58 kDa, 8.6 μM stock concentration) were immobilized onto the CM5 chip. AJ201 (3328.8 Da, 10 mM stock concentration) and AJ210 (3072.5 Da, 10 mM stock concentration) were used as analyte to flow over the ligand immobilized surface. Flow Cell (FC) 1 was used as the reference for FC2, FC3 for FC4. Anti-His antibody (2 mg/ml stock concentration) was diluted (1:100 dilution, 0.02 mg/ml diluted concentration) in 10 mM sodium acetate buffer at pH 4.5 and immobilized on all FCs to a level of ~8000 to 11000 RU using the standard amine coupling chemistry. HBS-P (10 mM HEPES pH 7.4, 150 mM NaCl, 0.05% v/v surfactant P20) was used as the immobilization running buffer. Human EphA2 was diluted (1:5 dilution) in HBS-P captured onto FC2 to a level of ~750 RU. Mouse EphA2 was diluted (1:10 dilution) in HBS-P captured onto FC4 to a level of ~750 RU. HBS-P was used as the capture running buffer. Based on these captured response values, theoretical R_max_ values were calculated and are presented in **Table S1**. The R_max_ values assume 1:1 interaction mechanism. Overnight kinetics were performed for all analytes in the presence of HBS-P+0.1% DMSO. The flow rate of all analyte solutions was maintained at 50 μL/min. The contact and dissociation times used were 120s and 600s, respectively. Glycine solution (pH 1.5) was injected for 15 s for surface regeneration and fresh ligands were captured in the beginning of each injection cycle. Sensorgrams from the overnight kinetics was evaluated by using 1:1 kinetics model fitting.

### Synthesis of [^nat^Ga]AJ201

In a reaction vial, to a stirred solution of AJ201 (0.6 mg, 0.18 µmol) in 200 µL of 1M sodium acetate buffer (NaOAc pH 5.0), 20 µL of aqueous 0.1M ^nat^GaCl_3_ solution and 0.6 mL of 0.1 M HCl was added. The reaction mixture was incubated at 65 °C for 30 min and purified on a RP-HPLC system using the same conditions as described in the synthesis of AJ210. The product [^nat^Ga]AJ201 was collected at RT 10.3 min, lyophilized to form an off-white powder (85% yield) and characterized by MALDI-TOF-MS (**Figure S2**). Theoretical chemical formula: C_143_H_224_GaN_45_O_41_S_3_; Exact Mass: 3392.5; Molecular Weight: 3395.5; and the observed MALDI-TOF-MS mass [M + H]^+^ is 3395.7.

### Synthesis of [^68^Ga]AJ201

^68^Ge/68Ga generator was eluted manually using 6 mL 0.1M HCl (Ultrapure trace-metal-free) in four different fractions (2 mL, 1 mL, 1 mL and 2 mL). To the microcentrifuge vial (1.5 mL) containing 200 μL of 1 M NaOAc buffer (pH 5) and 20 µg (6 nmol) of AJ201, added 4-6 mCi of ^68^GaCl_3_ in 0.6 mL from second fraction. The reaction mixture was incubated for 10 min at 65 °C and purified on a RP-HPLC system using the same conditions as described in the synthesis of AJ210. The radiolabeled product [^68^Ga]AJ201 was collected at RT 10.3 min, with decay corrected radiochemical yield of 66 ± 25 % (n = 53). Desired radiolabeled fraction was concentrated, formulated in 10% EtOH in saline and used for *in vitro* and *in vivo* studies. The whole radiolabeling process was done in ~35 min and quality control, stability studies and chemical identity was performed using the RP-HPLC system.

### Synthesis of [^68^Ga]AJ210

To the glass vial containing 200 μL of 1 M NaOAc buffer (pH 5) and 20 µg (6 nmol) of AJ210, added 4-6 mCi of ^68^GaCl_3_. The reaction mixture was incubated for 10 min at 95 °C and purified on a RP-HPLC system using the same conditions as described in the synthesis of AJ210. The radiolabeled product [^68^Ga]AJ210 was collected at RT 10.0 min. Desired fraction was concentrated, formulated in 10% EtOH in saline and used for *in vitro* and *in vivo* studies.

### Synthesis of [^225^Ac]AJ210

Ac-225 labeling was conducted using a reported method with modifications 51. Briefly, ^225^Ac(NO_3_)_3_ received as a solid and dissolved in 20 µL of 0.2 M HCl, was further diluted with 100 µL of 1 M sodium acetate buffer (NaOAc; pH = 5). To the microcentrifuge vial (1.5 mL) containing 50 μL of 1 M sodium acetate buffer (NaOAc; pH = 5), 1.8 mg of ascorbic acid and 40 µg (20 nmol) of AJ210, added 100 µCi of ^225^Ac(OAc)_3_ in 50 µL of 1 M NaOAc (pH = 5). The reaction mixture was incubated for 40 min at 95 °C, diluted with 500 µL of EtOH: saline (1:1) and purified on a RP-HPLC system. The radiolabeled product [^225^Ac]AJ210 was collected in saline containing 10 mg of ascorbic acid at RT 9.96 min with 40 % radiochemical yield. Desired radiolabeled fraction was concentrated, formulated in 10% EtOH in saline containing 10 mg of ascorbic acid and used for radiotherapy studies.

### *In vitro* stability of radiotracers

The stability of the radiotracers was assessed in saline and human serum at 37 °C to mimic physiological conditions. Approximately 1.5 mCi of the radiotracer, prepared in 100-150 µL of saline, was added to 0.5 mL of either saline or human serum in separate microcentrifuge tubes. The mixtures were gently vortexed and then incubated at 37 °C in an incubator. At predetermined time-points (1, 2, and 3 h), 100 µL aliquots were collected from the incubation mixtures for analysis. For serum samples, proteins were removed by adding 100 µL of saline and 50 µL of ethanol, followed by ultrafiltration using 10 kDa centrifugal filters made of regenerative cellulose membranes. The filtration process was performed by centrifugation at 14,000 rpm for 10 min and repeated once more to ensure complete protein removal. The filtrate was collected for analysis. The radiochemical integrity of the radiotracer in both saline and serum was evaluated using radio-RP-HPLC.

### Cell culture

Cell lines were either bought from ATCC or gifted from the collaborator. All cells were cultured in the recommended media in an incubator at 37 °C in an atmosphere containing 5% CO_2_ with humidity. Briefly, Panc1 and Hs766T were maintained in DMEM medium, CFPAC was maintained in IMDM media, AsPC1, BxPC3, Panc1005 and Su8686 in RPMI-1640. All cells were supplemented with 10% Fetal Bovine Serum and 1% P/S antibiotics.

### RNA extraction, cDNA synthesis and quantitative PCR

All cultured cell lines after removing the media, washed with cold PBS followed by snap-freezing in liquid nitrogen, stored at -80 C till further use. Total RNA was isolated using the RNAeasy Mini kit (Qiagen, Cat # 74104) following manufacturer's instructions. First-strand cDNA was synthesized from 1 μg of total RNA using the QuantiTect Reverse Transcription kit (Qiagen Cat.# 205311) following the standard protocol. The resulting cDNA was analyzed for the expression of EPhA2 gene using SYBR green in Applied Biosystems PCR machine. β-Actin, a housekeeping gene was used as an internal loading control. Primer sequences (5'-3') for EphA2 are: Fwd-TGTGCCAGGCAGGCTACG, Rev-CTCCAAGCAGGGGCTCTCA; and β-Actin Fwd-CACGAAACTACCTTCAACTCC Rev-CATACTCCTGCTTGCTGATCPCR. PCR cycle parameters involved an initial denaturation at 95 °C for 5 min, then 40 cycles of 10 s at 95 °C, 20 s at 58 °C. Fluorescence readings were taken at 75 °C after each cycle. The expression data was analyzed by normalize the Ct value with β-Actin and relative abundance was compared.

### Detection of EphA2 expression by flow cytometry

To evaluate EphA2 cell surface expression, human pancreatic cancer cells (1x10^6^) were stained with PE-labeled anti-human EphA2 monoclonal antibody (Clone SHM16, BioLegend Cat # 356804). Similarly mouse pancreatic cell lines were stained with FITC labeled anti-mouse EphA2 monoclonal antibody (SinoBiological Cat # 50586-R301-F). These samples were incubated in FACS buffer (0.5% FBS with 2 mM EDTA) for 30 min on ice. Samples were washed with 1X PBS and resuspended in 400 µL FACS buffer and analyzed on FACSCalibur flow cytometer. Data analysis was performed using FlowJo software for geometric mean fluorescence intensity (MFI) and histograms. Receptor density measurements were performed using Quantibrite Beads (BD Biosciences, cat #340495), which contain four levels of phycoerythrin (PE) per bead. Gates were drawn on Low, Medium Low, Medium High, and High PE binding beads, and the geometric mean (FL-2 A: PE-A) from these populations was correlated with the lot-specific PE molecule/bead on a logarithmic scale. This correlation was used to translate cell population geometric mean to receptor/cell for the respective cell type.

### *In vitro* binding assays with Ga-68 labeled radiotracers

*In vitro* binding assays were performed to determine the binding of [^68^Ga]AJ201 and [^68^Ga]AJ210 to Jurkat and PDAC cell lines. Approximately 1 μCi of radiotracer was incubated with cells in 100 μL culture media for 60 min at 4 °C. After incubation, cells were washed three times with ice cold PBS and counted on an automated gamma counter (1282 Compugamma CS, Pharmacia/LKBNuclear, Inc., Gaithersburg, MD). To demonstrate EphA2-specific binding of [^68^Ga]AJ201, blocking was performed with 2 µM of AJ201 and for [^68^Ga]AJ210, blocking was performed with 2 µM of AJ210. For kinetic study, 8×10^6^ Panc1 cells/vial were incubated with approximately 8 μCi/vial of [^68^Ga]AJ201 in culture media in triplicates at 37 °C and 4 °C in separate tubes. 1×10^6^ cells were aliquoted at predetermined timepoints (5, 15, 30, 60 min). Excess radioactivity was removed by washing three times with PBS. Activity in cell pellets were measured on an automated gamma counter.

### Tumor models

Animal studies were performed under Johns Hopkins University Animal Care and Use Committee (ACUC)-approved protocol (Principal investigator: Sridhar Nimmagadda, Ph.D. and Protocol number M021M175). Male and Female NSG mice (5-6 weeks old) were purchased from JHU immunocompromised core and used to establish xenografts by administering cells subcutaneously (top right flank, unless otherwise noted) to form various tumor models within 3-4 weeks. Following cell numbers were used: Panc1, AsPC1, BxPC3 (1 M), Hs766T and SU8686 (2 M), CFPAC and Panc1005 (5 M). Imaging or biodistribution studies (n = 3-5) were conducted on mice with tumor volumes of 100-200 mm^3^. The Panc1 orthotopic tumor model was established by surgically implanting a 3-5 mm^3^ section of Panc1 tumor xenograft directly onto the pancreas. The implanted tumors were allowed to grow and were ready for imaging after 10 days of implantation. At this stage, MRI measurements indicated that tumor sizes ranged from 10-50 mm^3^.

### Evaluation of pharmacokinetics of [^68^Ga]AJ201

Dynamic PET images were acquired on a Simultaneous 7T Bruker PET-MR scanner to evaluate the pharmacokinetics of [^68^Ga]AJ201. Mice bearing Panc1 tumor xenografts were anesthetized under 2.5% isoflurane and a catheter was fixed in the tail vein before being secured on the PET-MR bed. An activity of ~250 µCi (9.3 MBq; 0.5 to 0.8 nmol) of [^68^Ga]AJ201 was administered intravenously and whole-body PET dynamic scans were performed. Dynamic PET scans were initiated one min before radiotracer injection and acquired at varying intervals to capture early and late-phase kinetics: every 30 s from -1 to 5 min, every min from 5 to 15 min, every 3 min from 15 to 30 min, every 5 min from 30 to 60 min, and every 10 min from 60 to 90 min. The acquired PET data were reconstructed and corrected for radioactive decay and dead time using ParaVision 360 V2 by Bruker. The percentage of injected dose per cc (%ID/cc) values were obtained by drawing ROI on the tumor, muscle, heart, liver and kidney using PMOD software, and image fusion and visualization were also performed using PMOD software.

### Whole body PET-CT imaging of mouse xenografts

Mice bearing flank tumors were injected intravenously with ~250 µCi (9.3 MBq) of Ga-68 labeled radiotracer in 200 µL of 10% ethanol in saline. Mice were anesthetized under 2.5% isofluorane just before the PET imaging study and continued during the acquisition. PET images were acquired 60 min after injection of the radiotracer. Images were acquired in 2 bed positions for a total of 10 min using an ARGUS small-animal PET/CT scanner. Images were reconstructed using 2D-OSEM and corrected for radioactive decay and dead time. Image fusion, visualization, and 3D rendering were accomplished using Amira 2020.3.1 (FEI, Hillsboro, OR).

### *Ex vivo* biodistribution

Mice with 100-200 mm^3^ tumor volume were used for *ex vivo* biodistribution studies. For radiotracer pharmacokinetics and dosimetry studies, mice with Panc1 tumor xenograft received ~80 µCi (2.96 MBq) [^68^Ga]AJ201 and were sacrificed at pre-determined time points (5, 30, 60, 90 and 120 min). All other mice were injected with ~50 µCi (1.85 MBq; 0.2 to 0.5 nmol) Ga-68 labeled radiotracer and were sacrificed at 60 min. The selected tissues were collected, weighed, counted, and their %ID/g values calculated for biodistribution analysis. For pharmacokinetics and dosimetry studies, selected tissues included blood, muscle, tumor, thymus, heart, lung, liver, pancreas, stomach, small intestine, large intestine, spleen, adrenals, kidney, bladder, femur, brain and tumor xenografts. For other studies, selected tissues were blood, muscle, tumor, heart, lung, liver, pancreas, small intestine, spleen, kidney.

### PET-MR imaging of Panc1 orthotopic tumor model

Mice having orthotopic panc1 tumors were injected intravenously with [^68^Ga]AJ201 and PET images were acquired at 60 min after radiotracer injection for 10 min in 1 bed positions (full body) and whole-body MRI using a Simultaneous 7T Bruker PET-MR. The PET data were acquired, reconstructed, and corrected for radioactive decay using ParaVision 360 V2 by Bruker. The %ID per cc values were obtained after drawing ROI (regain of interest) in tumor and muscle using PMOD software. Image fusion, visualization, were also accomplished using PMOD software (Bruker)**.**

### Immunohistochemistry of xenografts

To perform immunohistochemistry, the tissue slides were deparaffinized by baking at 60 °C and washing with xylene and alcohol. Antigen retrieval was carried out using citrate buffer (pH 6.0, 95-100 °C, 20 min) and the endogenous peroxidase and alkaline phosphatase activity was blocked using BioXALL. The primary anti-human EphA2 antibody was applied at a dilution of 1:250 and incubated overnight at 4 °C (Cell Signaling Cat# 6997S). After washing with PBS, the secondary antibody, Signalstain Boost IHC Detection Reagent (HRP), was applied and incubated for 30 min at room temperature. The slides were washed and developed using ImmPACT DAB substrate (Vector Lab #SK4105). After washing, the slides were counterstained with Mayer's Hematoxylin for 1 min, dehydrated using alcohol and xylene, and then cover slipped.

### Therapeutic evaluation of [^225^Ac]AJ210

Male C57BL/6 mice (5-6 weeks old) were used to establish syngeneic tumor by administering KPC cells subcutaneously in top right flank. For this study, 0.5 million cells were inoculated subcutaneously into the upper flank of each animal. Tumor growth was closely monitored, and once tumors became palpable, their volumes were measured using a vernier caliper. Animals were then randomized into groups to ensure balanced baseline tumor sizes, with median volumes of approximately 60 mm^3^ (PBS group median: 60 mm^3^; radiotherapeutic group median: 57 mm^3^). Following randomization, two subsequent doses of 0.5 µCi of [^225^Ac]AJ210 were administered intravenously with the interval of 24 h. Similarly, saline was administered in the control group. Measurements of tumor volume and body weight were conducted in alternative days until the end of study. Blood was collected retro-orbitally at 3-, 7- and 28-days post radiotherapy injection and hematological parameter were assessed using SCIL Vet ABC Plus instrument.

### H&E staining

Tissue blocks and slides of liver, kidneys and lungs were prepared by JHU histology core and staining was also carried out by the Core. Briefly, tissue samples were fixed in formalin for 24 h and rinsed with PBS and stored in PBS. These tissue samples were dehydrated through graded ethanol series, cleared in xylene, and embedded in paraffin. Sections (4-5 µm thick) were cut using a microtome and mounted on glass slides. The slides were deparaffinized in xylene, rehydrated through graded ethanol to distilled water, and stained with hematoxylin for 5 min. After rinsing in running water, sections were differentiated in 1% acid alcohol, blued in ammonia water, and counterstained with eosin for 1-2 min. Slides were dehydrated, cleared in xylene, and mounted with a coverslip using a permanent mounting medium. Images were captured under a brightfield microscope to evaluate tissue structure and histopathological features.

### Statistical analysis

All statistical analyses were performed using Prism 9.0 Software (GraphPad Software, La Jolla, CA). Unpaired Student's *t test* and one- or two-way ANOVA were utilized for column, multiple column, and grouped analyses, respectively. Statistical significance was set at ns, *P* ≥ 0.05; *, *P* ≤ 0.05; **, *P* ≤ 0.01; ***, *P* ≤ 0.001; ****, *P* ≤ 0.0001. Correlation was performed using simple linear regression without keeping the term constant at zero.

## Results

### Analysis of EphA2 expression in CCLE and TCGA human genomic database

EphA2 plays a crucial role in cancer tumorigenesis and has been considered a potential therapeutic target 15. However, to fully harness its potential as an imaging and therapeutic target, a detailed and comparative understanding of EphA2 expression in both cancerous and healthy tissues is necessary.

**Figure 1:**
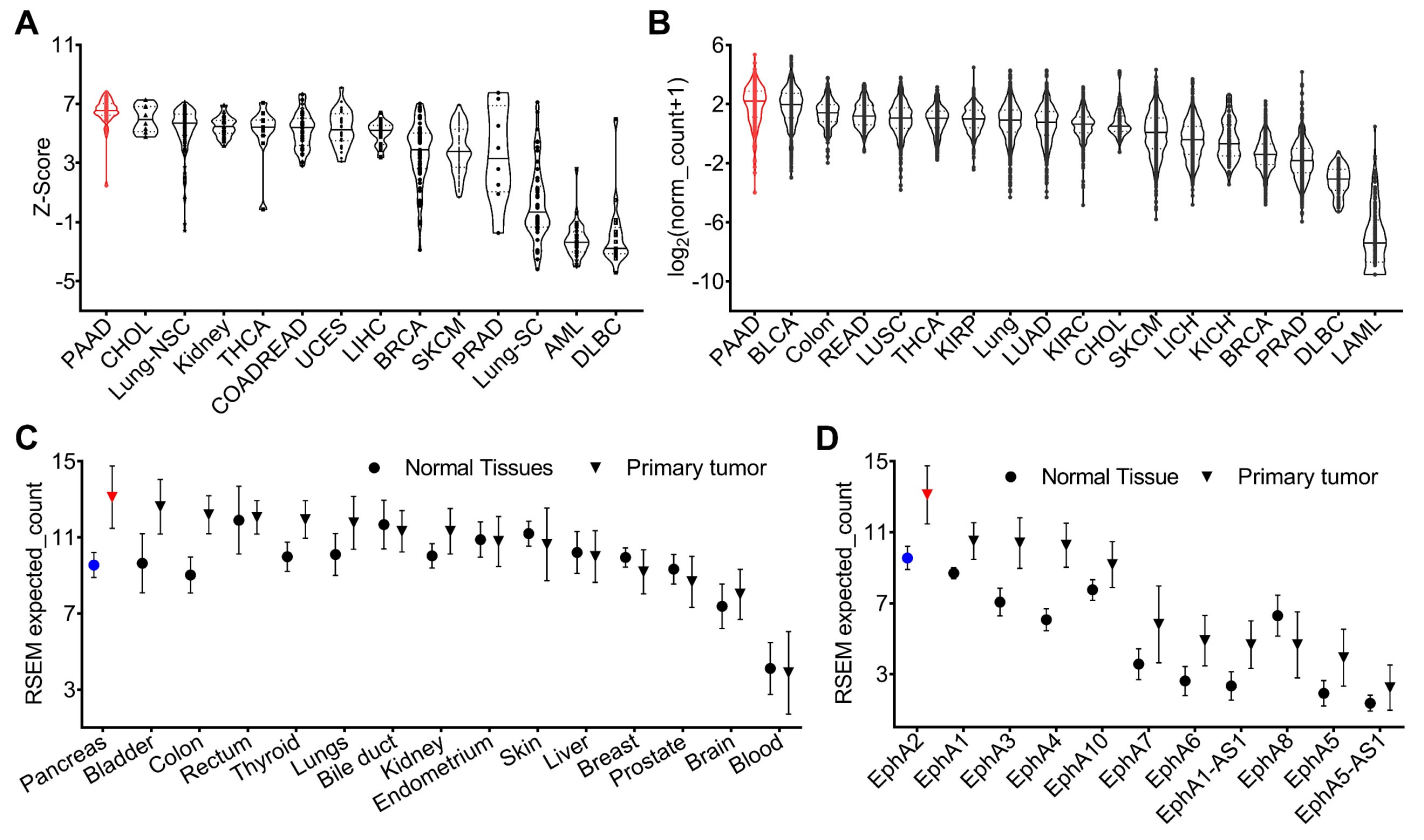
** CCLE and TCGA database of mRNA expression of EphA2. A)** EphA2 expression in cell lines in CCLE. **B)** EphA2 mRNA expression in different types of tumors in TCGA. **C)** Comparison of mRNA expression of EphA2 in different normal tissues and corresponding tumors. **D)** Expression of different EPH receptors in pancreatic tumors. PAAD = pancreatic cancer; BLCA = Bladder cancer; READ = Rectal cancer; LUSC = Lung squamous cell carcinoma; THCA = Thyroid cancer; KIRP = Kidney papillary cell carcinoma; LUAD = Lung adenocarcinoma; KIRC = Kidney clear cell carcinoma; CHOL = Bile duct cancer; SKCM = melanoma; LIHC = Liver cancer; KICH = Kidney chromophobe; BRCA = Breast cancer; PRAD = Prostate cancer; DLBC = Large-B-cell lymphoma; LAML = Acute myeloid leukemia.

An analysis of genomic data from the Cancer Cell Line Encyclopedia (CCLE) revealed variable expression of the EphA2 gene across different human cancer cell lines, with pancreatic cancer cells showing higher expression levels compared to others (**Figure 1A**). To validate the cell line data and characterize EphA2 expression in human cancers, we analyzed genomic data from The Cancer Genome Atlas (TCGA). This analysis showed variable expression of EphA2 across different human cancer types, with notably high expression in pancreatic, bladder, and colorectal cancers, and low expression in lung, breast, prostate, and lymphoma. The median expression of the EphA2 gene was particularly high in pancreatic cancer compared to other cancer types (**Figure 1B**). Furthermore, EphA2 expression was consistently higher in tumors compared to their corresponding normal tissues, with the most significant difference observed between PDAC and normal pancreas tissue (**Figure 1C**). To confirm the database mining results, we further analyzed PDAC samples in a TMA by IHC analysis and found that more than 95% of tumor samples stained for membranous EphA2 expression (**Figure S1**).

Within the Ephrin receptor family, nine different functional receptors (EphA1-EphA9) are known in the mouse and human genomes. To understand the expression of different Ephrin receptors in PDAC, we analyzed the TCGA dataset and compared the expression of different Ephrin receptors in PDAC and healthy pancreas tissue. Our analysis revealed greater expression of EphA2 in PDAC compared to other Eph receptors (**Figure 1D**). Collectively, these genomic analyses highlight the high expression pattern of EphA2 in PDAC, which may provide a robust platform for the development of radiotheranostic agents.

### Structure and evaluation of AJ201 for EphA2 receptor binding

**Figure 2:**
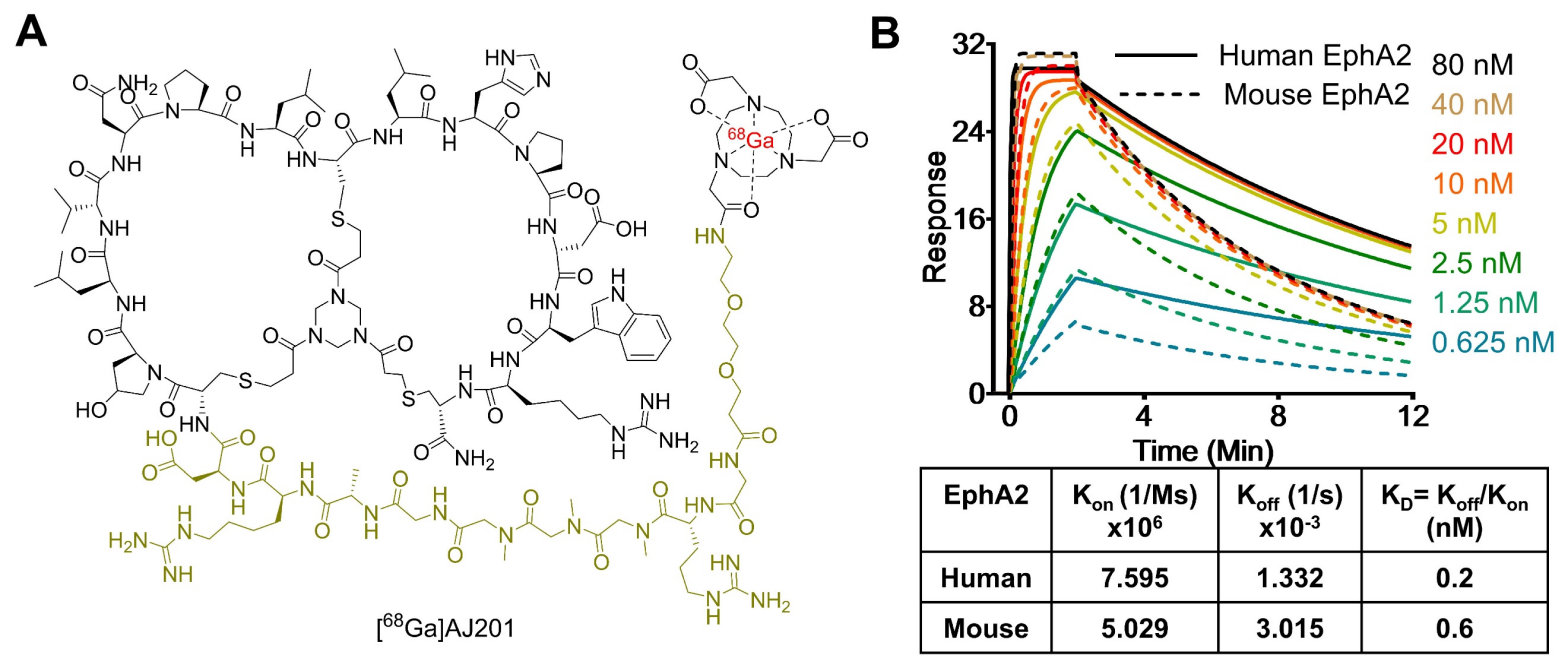
**Structure and *in vitro* characterization of AJ201. A)** Structure of bicyclic peptide AJ201 having NOTA as bifunctional chelator for ^68^Ga-labeling. In the structure, black color represents binding moiety, parakeet color represents the linker and red color represents the radiometal **B)** Surface plasmon resonance (SPR) analysis showing affinity of AJ201 for EphA2 using recombinant human (solid) and mouse (dashed) EphA2 proteins.

Recently, low molecular weight peptides that bind EphA2 have been reported. Here, we introduce AJ201, a 15-amino acid bicyclic peptide derived from BCY6099, comprising six hydrophilic and nine hydrophobic amino acids 17, 18. To enhance its hydrophilicity, we incorporated a linker with a bis-ethylene glycol moiety and three sarcosine amino acid chain, whereas BCY6099 utilizes a linker composed of 10 sarcosine units. Since peptides often undergo renal clearance, we have further modified AJ201 by incorporating an arginine and a glycine motif, a known brush border membrane cleavable linker, with the goal of reducing renal retention of radioactivity. To facilitate radiotracer generation, the modified 24-amino acid peptide was then conjugated with bifunctional chelators, NOTA (1,4,7-Triazacyclononane-1,4,7-triacetic acid) (**Figure 2A**) and DOTA (2,2',2'',2'''-(1,4,7,10-tetraazacyclododecane-1,4,7,10-tetrayl)tetraacetic acid) and characterized by mass spectrometry (**Figure S2**). NOTA is extensively used to form a highly stable complex with the radiometal [^68^Ga], while DOTA serves as a chelator for both [^68^Ga] and [^177^Lu] the latter being part of an FDA-approved radiotheranostic pair 27.

To assess the affinity of AJ201 for EphA2, we conducted surface plasmon resonance (SPR) assays (**Table S1**). The results showed that AJ201 binds to both human and mouse EphA2 with strong affinity, displaying dissociation constants (KD) of 0.2 nM and 0.6 nM, respectively (**Figure 2B**). These findings suggest that AJ201 is a promising candidate for further development as a molecular imaging agent for tumor detection.

### Evaluation of EphA2 receptor expression in PDAC cell lines by RT-PCR and flow cytometry

**Figure 3:**
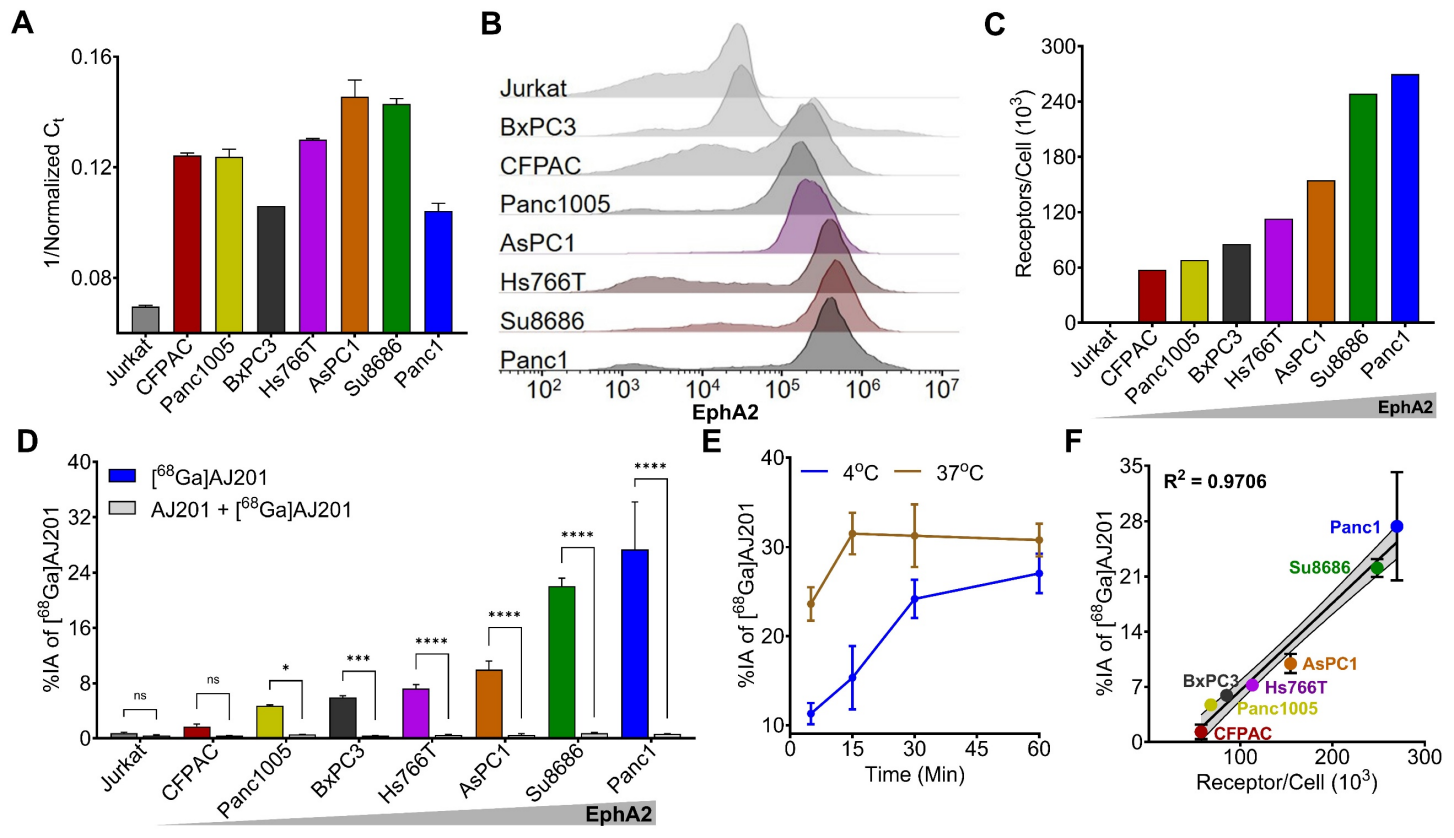
***In vitro* specificity of [^68^Ga]AJ201 for EphA2 in human pancreatic cancer cells. A)** Quantification of EphA2-mRNA using RT-PCR. **B)** Flow cytometry analysis of EphA2 receptor expression on cell surface. **C)** A representative plot of EphA2 receptor density in PDAC cells measured by quantibrite assay. **D)** [^68^Ga]AJ201 binding (percent incubated activity, %IA) to different cells. Cells were incubated with 1 µCi [^68^Ga]AJ201 at 4 °C for 1 h. [^68^Ga]AJ201 uptake is EphA2 expression dependent, and co-incubation with 2 μM of non-radioactive AJ201 (0.2 nmol, blocking dose) significantly reduced radiotracer uptake confirming EphA2 specificity. **E)** *In vitro* uptake of [^68^Ga]AJ201 over 60 min in Panc1 cells at 4 ˚C and 37 ˚C. **F)** Correlation of [^68^Ga]AJ201 uptake with surface EphA2 receptor density. Data in panels **A**, **D**, **E** and **F** are presented as mean ± SD (n = 3-4). Significance was calculated using multiple unpaired *t* test in **D**; ns, *P* ≥ 0.05; *, *P* ≤ 0.05; ***, *P* ≤ 0.001; ****, *P* ≤ 0.0001. Simple linear regression and Pearson coefficient were used in **F**. All *in vitro* experiments were performed three times and representatives are shown.

To confirm the observations from the CCLE dataset, we selected seven human PDAC cell lines (CFPAC1, Panc1005, BxPC3, Hs766T, AsPC1, Su8686, and Panc1) and evaluated EphA2 expression using RT-PCR and flow cytometry. Jurkat cells, a human T lymphocyte cell line, served as a negative control. RT-PCR analysis revealed high and variable expression levels of EphA2 mRNA in all the PDAC cell lines tested, while Jurkat cells showed only basal expression (**Figure 3A**). These findings were corroborated by flow cytometry, which assessed cell surface EphA2 expression. High and variable levels of cell surface EphA2 were detected across all PDAC cell lines, with the highest expression in Panc1 cells and the lowest in Jurkat cells (**Figure 3B**).

Further analysis of receptor density in PDAC cells using PE-Quantibrite beads demonstrated significant variability. Panc1 cells exhibited the highest receptor density (2.7 x 10^5^ receptors per cell), while CFPAC1 cells had the lowest (0.58 x 10^5^ receptors per cell) (**Figure 3C**). Other PDAC cell lines showed the following receptor densities: Panc1005 (0.68 x 10^5^), BxPC3 (0.85 x 10^5^), HS766T (1.3 x 10^5^), AsPC1 (1.55 x 10^5^), and Su8686 (2.48 x 10^5^).

Interestingly, we did not observe a clear positive relationship between mRNA abundance and receptor density in PDAC cells. This discrepancy underscores the importance of validating EphA2 expression through multiple methods to complement genomic data and better understand the activity of EphA2-targeted therapeutics.

### Radiolabeling and *in vitro* evaluation of [^68^Ga]AJ201

To develop a PET imaging agent, AJ201 was labeled with [^68^Ga], achieving high radiochemical yields (66 ± 25%, n = 53, decay-corrected), radiochemical purity (>98%), moderate specific activity (15-20 GBq/µmol) and co-eluted with [^nat^Ga]AJ201, confirming its chemical identity (**Scheme S2, Figure S3**). [^68^Ga]AJ201 demonstrated excellent stability at 37 °C for at least 3 h in formulation buffer (10% EtOH/saline) and human serum (**Figure S4**).

We conducted cell binding assays to assess the *in vitro* specificity of [^68^Ga]AJ201 for EphA2. Seven PDAC cell lines were incubated with 1 µCi [^68^Ga]AJ201 for 60 min, followed by washing and measurement of cell-associated activity. All steps were done at 4 °C to minimize internalization. We observed variable uptake of [^68^Ga]AJ201, with the highest uptake in Panc1 cells (27.4 ± 6.8 percent incubated activity, %IA) and the lowest in CFPAC cells (1.7 ± 0.3 %IA). The uptake order was: Panc1 (27.4 ± 6.8 %IA) > Su8686 (22.0 ± 1.1 %IA) > AsPC1 (9.9 ± 1.2 %IA) > Hs766T (7.2 ± 0.6 %IA) > BxPC3 (5.9 ± 0.2 %IA) > Panc1005 (4.7 ± 0.1 %IA) > CFPAC (1.7 ± 0.3 %IA). The least radioactivity was bound to Jurkat cells (0.8 ± 0.1 %IA), which showed basal EphA2 expression (**Figure 3D**). These results indicate that [^68^Ga]AJ201 binding to PDAC cells is dependent on EphA2 expression on the cell surface.

To understand binding kinetics, Panc1 cells were incubated with [^68^Ga]AJ201 at 4 °C and 37 °C for different time intervals (**Figure 3E**). We observed rapid uptake of radioactivity by Panc1 cells, reaching a plateau within 60 min. At 37 °C, uptake was fast, reaching equilibrium by 15 min, with a slight decline between 15 and 60 min. In contrast, at 4 °C, uptake was slower, stabilizing around 30 min without a subsequent decrease. These differences likely reflect faster binding and internalization at 37 °C, with a greater overall magnitude, indicating that [^68^Ga]AJ201 is rapidly internalized upon binding to EphA2. To confirm the specificity of [^68^Ga]AJ201 binding to EphA2, we conducted additional experiments. Binding of [^68^Ga]AJ201 to Panc1 cells was significantly reduced by more than 95% (P<0.0001) in the presence of non-radioactive AJ201 (2 µM), confirming that [^68^Ga]AJ201 binding is specific to EphA2 (**Figure 3D**). Further validation involved correlating [^68^Ga]AJ201 uptake with receptor density quantified using Quantibrite beads. The results showed a strong correlation between radiotracer uptake and receptor density (R^2^ = 0.97, P<0.0001) (**Figure 3F**). These findings collectively demonstrate that the *in vitro* binding of [^68^Ga]AJ201 to PDAC cells is directly dependent on surface expression of EphA2.

### *In vivo* distribution of radiotracer in mice bearing human tumor xenografts

**Figure 4:**
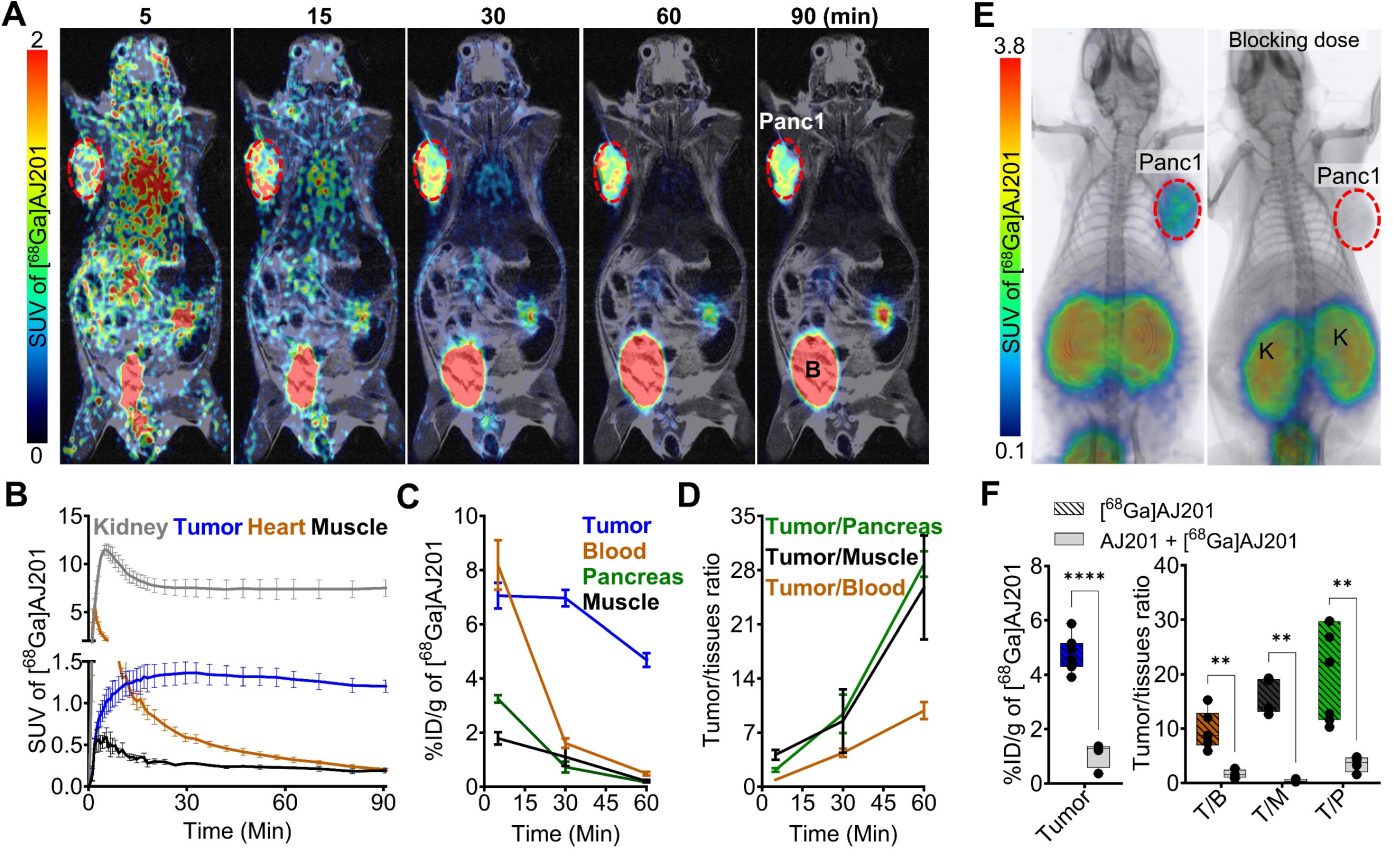
**Pharmacokinetics of [^68^Ga]AJ201 in NSG mice bearing Panc1 tumor xenografts. A)** Coronal sections of the fused dynamic PET/MR images showing [^68^Ga]AJ201 distribution. Primary tumor is indicated by red circle. Mice were intravenously injected with ~ 9.25 MBq (~250 µCi) [^68^Ga]AJ201; B, Bladder. **B)** Time-activity curves of [^68^Ga]AJ201 in the kidney, tumor, heart and muscle derived from PET data in A. **C)** Uptake of [^68^Ga]AJ201 in tumor, blood, pancreas and muscle derived from *ex vivo* biodistribution study (percent incubated dose per gram, %ID/g). **D)** Tumor-to-muscle, tumor-to-blood and tumor-to-pancreas ratios derived from biodistribution data. **E)** Whole-body PET/CT images of Panc1 xenografts with [^68^Ga]AJ201, without (left) and with (right) pre-administration of a blocking dose (50 µg of AJ201) (tumor denoted with dashed red line and K = Kidney). **F)** [^68^Ga]AJ201 quantification in tumors by *ex vivo* biodistribution in mice treated with and without a blocking dose where T/B is tumor-to-blood; T/M is tumor-to-muscle and T/P is tumor-to-pancreas ratios; data in panels **B** represented as mean ± SEM (n = 3); data in panels **C** and **D** are from mice intravenously injected with ~ 2.96 MBq (~80 µCi) [^68^Ga]AJ201 and sacrificed at different time-points after injection, data represented as mean ± SEM (n = 3 or 4); data in figure **F** is shown as box and whisker plots (median ± IQR) showing all data points (n = 4). Statistics were calculated using multiple unpaired *t* test in **F**. ** *P* ≤ 0.01; ****, *P* ≤ 0.0001.

To evaluate the pharmacokinetics of [^68^Ga]AJ201, we conducted whole-body dynamic PET scans on mice with Panc1 tumor xenografts. [^68^Ga]AJ201 uptake was observed in Panc1 tumors within 5 min post-administration (**Figure 4A**). High accumulation of radioactivity in the Panc1 tumors and washout from healthy tissues resulted in high-contrast tumor-specific images by 60 min. However, high radioactivity accumulation was also observed in the kidneys. Region of Interest (ROI) analysis indicated continuous accumulation of radioactivity in tumors, peaking at 30 min and remaining constant until 90 min. In contrast, there was continuous washout of radioactivity from non-target tissues such as muscle (**Figure 4B**). Collectively, these data indicate that [^68^Ga]AJ201 exhibits optimal pharmacokinetics and biodistribution, providing high-contrast images of PDAC within 60 min post-administration.

To validate the imaging data, radiotracer uptake in different tissues was measured at pre-determined time points by *ex vivo* biodistribution studies in mice with Panc1 tumors. These studies showed high radioactivity accumulation in tumors, kidneys, and bladder at all time points and radioactivity in the kidneys remined high until 120 min. Tumors exhibited high radioactivity accumulation (4.7-7.9 percent incubated dose per gram, %ID/g) between 5 and 120 min post-administration. In contrast, low radioactivity accumulation was observed in other tissues such as the heart (0.29 ± 0.08 %ID/g), lung (0.65 ± 0.04 %ID/g), liver (0.32 ± 0.02 %ID/g), intestines (0.49 ± 0.07 %ID/g), femur (0.28 ± 0.06 %ID/g), and brain (0.04 ± 0.01 %ID/g). Rapid clearance of the radiotracer from blood and muscle contributed to high tumor-to-blood (9.9 ± 1.1) and tumor-to-muscle (25.8 ± 6.7) ratios by 60 min. Minimal accumulation of radioactivity in the normal pancreas resulted in high tumor-to-pancreas ratios (28.8 ± 0.9) (**Figure 4C, 4D, Table 1**), demonstrating the potential of [^68^Ga]AJ201 for visualizing PDAC. Next, to confirm the *in vivo* specificity of [^68^Ga]AJ201 for EphA2, non-radioactive AJ201 (1 mg/kg) was injected subcutaneously 30 min prior to [^68^Ga]AJ201 in mice with Panc1 tumors. PET/CT images and *ex vivo* biodistribution data showed a significant reduction in tumor radioactivity uptake, indicating that [^68^Ga]AJ201 uptake in Panc1 tumors is EphA2-specific (**Figure 4E and 4F**). Collectively, these *in vivo* data indicate that [^68^Ga]AJ201 exhibits suitable pharmacokinetics and image contrast for detecting PDAC.

**Figure 5:**
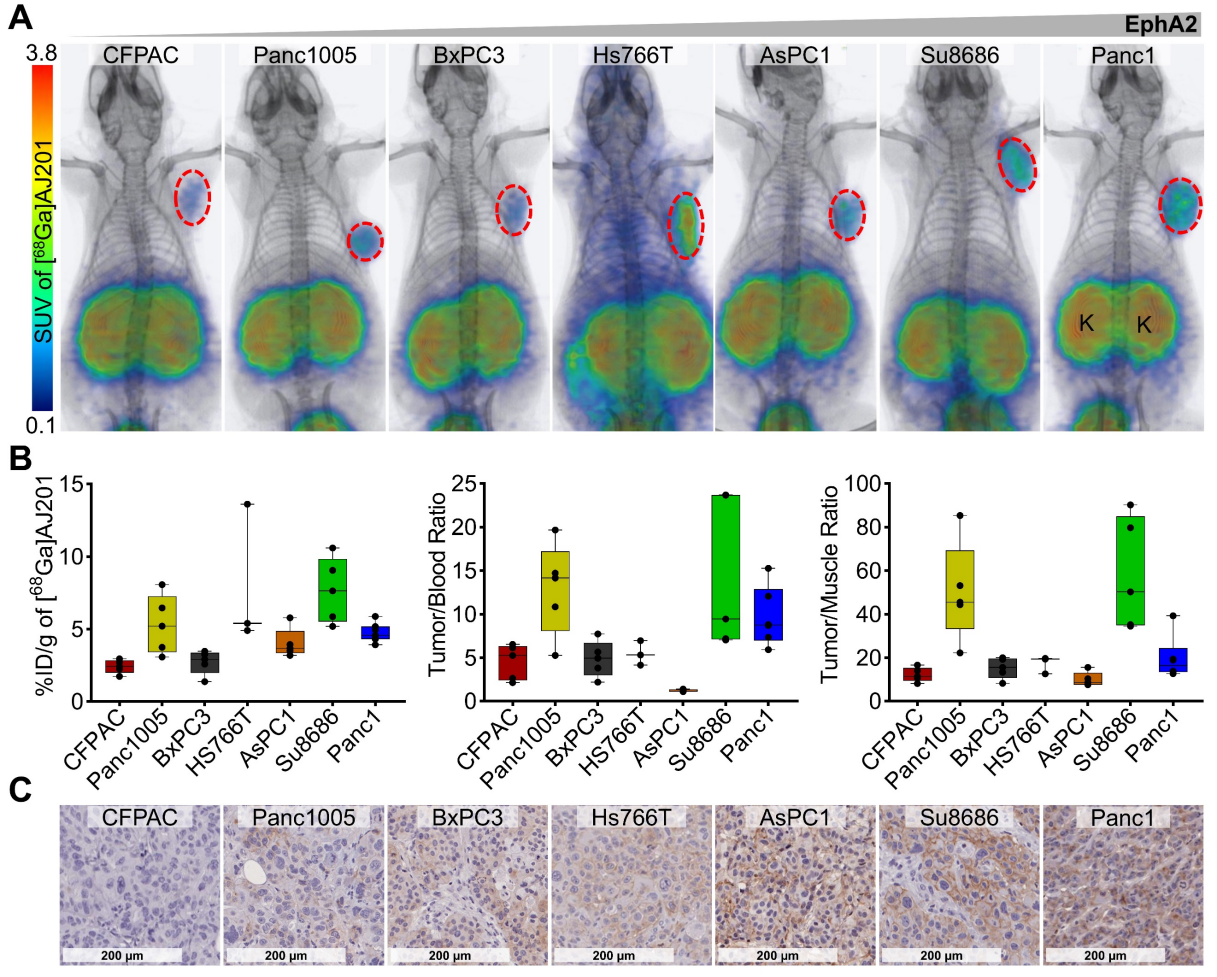
***In vivo* specificity of [^68^Ga]AJ201 for EphA2 in NSG mice with PDAC tumor xenografts. A)** Whole-body PET/CT images of different human PDAC xenografts at 60 min after the injection of radiotracer. Mice were injected with ~ 7.4 MBq (~200 µCi) [^68^Ga]AJ201; K, kidney. Panc1 mouse image is reproduced from figure 4E. **B)** [^68^Ga]AJ201 uptake quantification (%ID/g) in different PDAC tumors by *ex vivo* biodistribution at 60 min after injection. Left: %ID/g of different tumor xenografts, tumor-to-blood (middle) and tumor-to-muscle (right) ratios in each individual mice harboring respective xenografts.** C)** IHC staining for EphA2 expression in PDAC xenografts (digitally scanned at 40x).; data in figure **B** is shown as box and whisker plots (median ± IQR) showing all data points (n = 4-5).

To evaluate the potential of [^68^Ga]AJ201 to detect variable EphA2 levels *in vivo*, PET/CT images were acquired in six additional pancreatic tumor xenografts (CFPAC, Panc1005, BxPC3, AsPC1, Hs766T, and Su8686) at 60 min post-administration. We observed high and variable uptake of [^68^Ga]AJ201 in PDAC xenografts and minimal uptake in normal tissues across all models tested (**Figure 5A**). *Ex vivo* biodistribution studies in the same tumor models showed high uptake in Hs766T (8.0 ± 2.8 %ID/g) and Su8686 (7.7 ± 1.0 %ID/g) xenografts, moderate uptake in Panc1005 (5.3 ± 0.9 %ID/g) and AsPC1 (4.0 ± 0.5 %ID/g), and low uptake in CFPAC (2.4 ± 0.2 %ID/g) and BxPC3 (2.7 ± 0.4 %ID/g). These results support the imaging studies, showing minimal uptake in healthy tissues, except for kidneys, and high tumor-to-blood and tumor-to-muscle ratios in all xenograft models (**Figure 5B, Table S2**). Additionally, high tumor-to-pancreas ratios across all xenografts confirmed that [^68^Ga]AJ201 detects variable EphA2 levels in PDAC *in vivo* (**Figure S5**). Furthermore, IHC analysis of the same tumors showed high immunoreactivity in Panc1, Hs766T and Su8686, moderate immunoreactivity in Panc1005 and AsPC1, and low immunoreactivity in CFPAC and BxPC3 xenografts (**Figure 5C**). This result supports the observation that [^68^Ga]AJ201 uptake in imaging and biodistribution studies is indeed EphA2-specific. We generally observed that cell lines and tumors with high EphA2 expression exhibited higher [^68^Ga]AJ201 uptake, underscoring the robustness of our imaging agent. However, exceptions such as the Panc1005, Hs7667 and Su8686 models, which showed greater variability, may be attributed to unique tumor microenvironmental features specific to each model. These factors, including tumor perfusion, receptor accessibility, or stromal density, may not be fully captured by IHC. Collectively, imaging and biodistribution data across seven PDAC xenografts establish the potential of [^68^Ga]AJ201 to detect PDAC and variable EphA2 levels *in vivo*.

To investigate the *in vitro* and *in vivo* specificity of [^68^Ga]AJ201 for mouse EphA2, we conducted *in vitro* binding studies with the KPC, a mouse cell line, which exhibits high EphA2 expression, as confirmed by flow cytometry (**Figure S6A**). The *in vitro* binding study demonstrated EphA2-dependent uptake (**Figure S6B**). Further, [^68^Ga]AJ201-PET imaging and *ex vivo* biodistribution data confirmed the *in vivo* specificity of [^68^Ga]AJ201 for EphA2-expressing tumors (**Figure S6C-D, Table S3**). These results collectively suggest that [^68^Ga]AJ201 effectively targets EphA2-expressing cells in immunocompetent mice.

### Human radiation dosimetry estimates

Pharmacokinetic data obtained from biodistribution studies of Panc1 tumor-bearing mice were utilized to predict the time-integrated activity coefficients (TIACs, formerly known as residence times) of [^68^Ga]AJ201 in humans, as previously described 28-30. These TIAC estimates were then input into MIRDCalc to determine the absorbed dose coefficients for organs from ^68^Ga using the ICRP adult female reference phantom (**Table S4**). The results indicated that the kidneys received the highest absorbed dose (0.38 rem/mCi), followed by the bladder (0.15 rem/mCi), adrenals (0.06 rem/mCi), and lungs (0.04 rem/mCi). Based on these results, a 13 mCi dose can be safely administered for PET imaging, with an estimated effective dose equivalent of less than 5 rem.

### *In vivo* distribution of [^68^Ga]AJ201 in orthotopic models of PDAC

**Figure 6:**
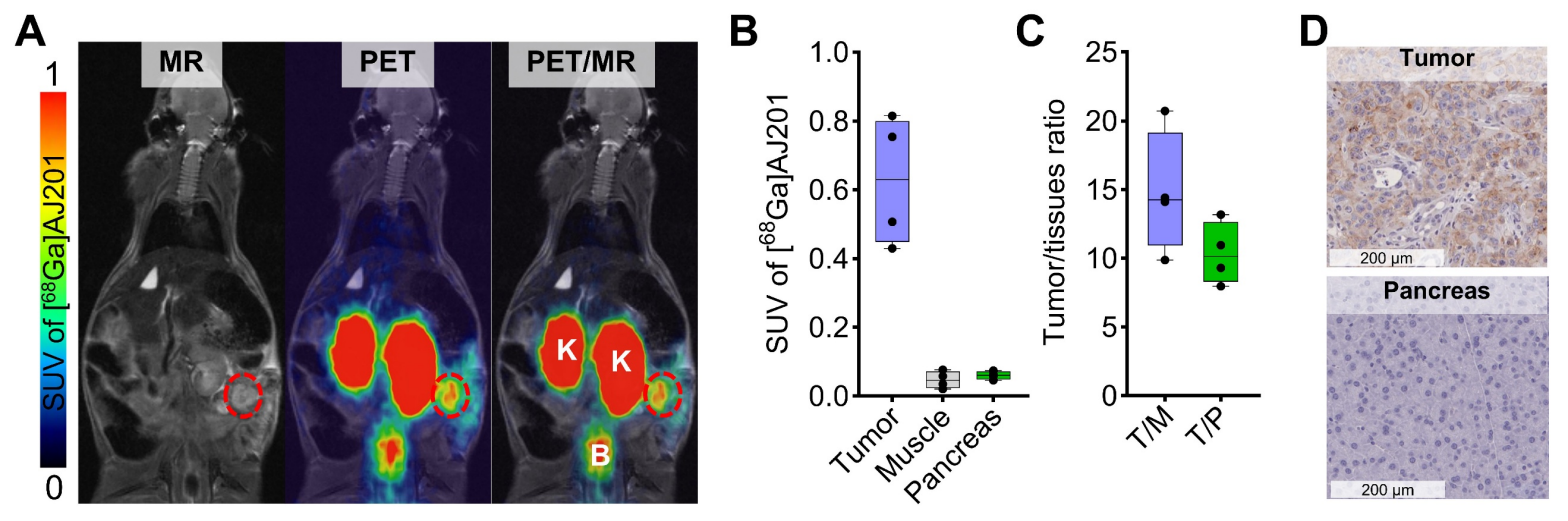
*** In vivo* distribution of [^68^Ga]AJ201 in Panc1-orthotopic tumor model. A)** Coronal sections of the fused PET/MR images showing [^68^Ga]AJ201 distribution. Primary tumor is indicated by red circle; K = Kidney, B = Bladder. **B)** Quantification of accumulated activity in tumor, muscle and pancreas of images shown in panel A (n = 4-5) **C)** tumor-to-muscle (T/M) and tumor-to-pancreas (T/P) ratios from the images shown in panel A (n = 4) **D)** EphA2 IHC of orthotopic Panc1 tumor and normal pancreas to validate the EphA2 expression. Panc1 orthotopic tumor model was generated by surgically implanting a 3-5 mm³ section of Panc1 tumor xenograft directly onto the pancreas. The implanted tumors were allowed to grow for 10 days, after which they were ready for imaging. At this stage, MRI measurements indicated that tumor sizes ranged from 10-50 mm^3^. data in figure **B** and **C** is shown as box and whisker plots (median ± IQR) showing all data points (n = 3-5).

Leveraging the favorable pharmacokinetics and high tumor-to-pancreas ratios observed, we assessed the potential of [^68^Ga]AJ201-PET for visualizing orthotopic pancreatic tumors. In orthotopic Panc1 tumor model, PET/MR images acquired 60 min after injecting [^68^Ga]AJ201 revealed a robust uptake of the tracer in the tumor, while minimal accumulation was observed in normal tissues (**Figure 6A and 6B**) with a high tumor-to-muscle ratios of 14.77 ± 4.46 and tumor-to-pancreas ratios of 10.35 ± 2.24 (**Figure 6C**). To further corroborate the imaging data, we conducted immunohistochemistry (IHC) analysis for EphA2 receptor expression on tumor sections (**Figure 6D**). The IHC results aligned with the imaging findings, providing additional validation. This successful demonstration of detecting small-sized orthotopic pancreatic tumors underscores the potential utility of [^68^Ga]AJ201-PET or its analogs for PDAC visualization.

### EphA2 targeted radiotherapeutic [^225^Ac]AJ210 controls tumor growth in syngeneic KPC tumor model

Next, we hypothesized that the consistently high expression of EphA2 observed in PDAC could be leveraged for targeted molecular radiotherapy. We chose the alpha-particle emitter [^225^Ac] for radiation delivery due to its ability to induce largely irreparable double-strand breaks, leading to selective cytotoxicity in cancer cells. This approach is increasingly recognized as a safe and effective treatment option 11, 31.

To test this hypothesis, we first developed a DOTA-conjugated derivative of AJ201, named AJ210, to facilitate the chelation of [^225^Ac]. The DOTA analog, AJ210, labeled with Ga-68 (**Scheme S3**), demonstrated *in vitro* affinity and specificity, high *in vitro* stability as well as favorable pharmacokinetics, tumor uptake, and retention properties similar to AJ201 in Panc1 and KPC tumor models (**Figure S7-S11, Table S5**). We then substituted [^225^Ac] for Ga-68 to study therapeutic effects in a KPC tumor model (**Figure S12**). The KPC model is a well-established tumor model that closely recapitulates the aggressive, treatment-resistant nature and desmoplastic stroma of human PDAC, making it invaluable for studying disease progression and testing novel therapeutic approaches.

**Figure 7:**
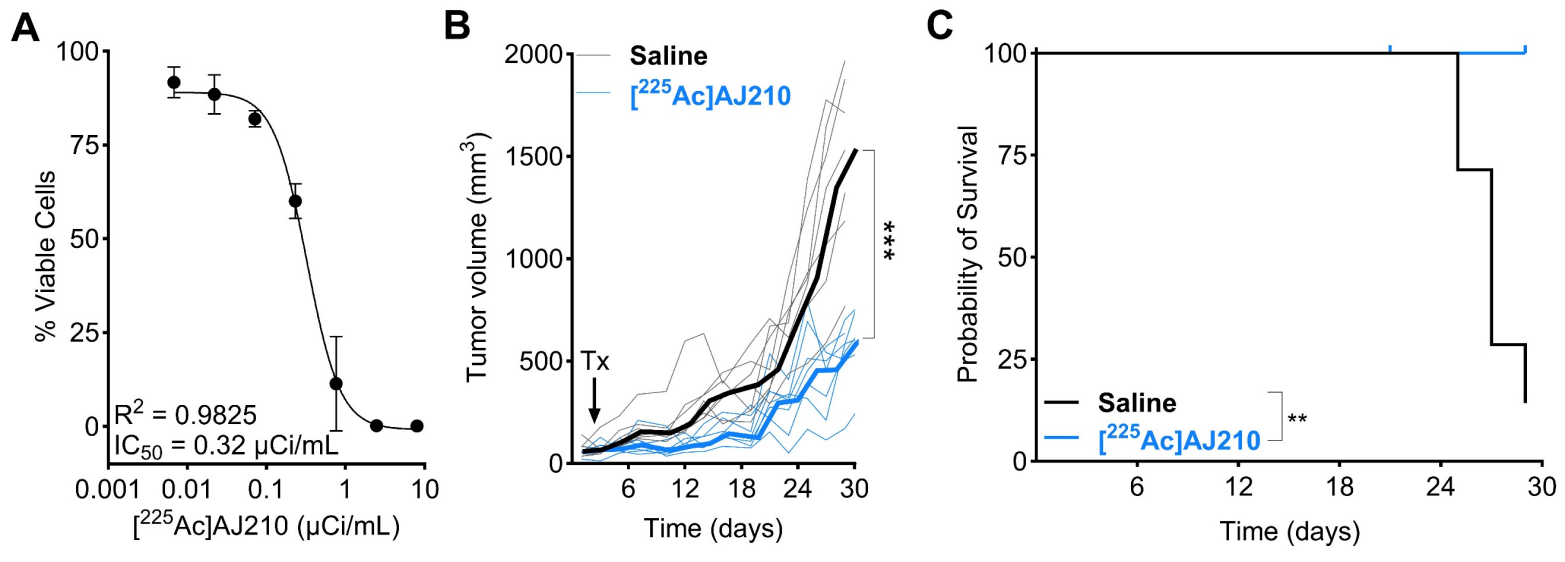
***In vitro* and *in vivo* therapeutic efficacy of [^225^Ac]AJ210 A)** *In vitro* cell growth inhibition by [^225^Ac]AJ210 after 72 h incubation with KPC cells (data; mean ± SD, n = 4) at 37 °C. **B)** Effect of [^225^Ac]AJ210 (n = 7) on the KPC tumor growth (data; median bold line and individual replicates) after administration of total 37 kBq via tail-vein injection; each line represents one animal, arrow indicates [^225^Ac]AJ210 treatment (Tx) **C)** Kaplan-Meier plot of survival for the group treated with saline and [^225^Ac]AJ210. Median survival of saline treated group is 27 days and [^225^Ac]AJ210-treated group did not reach. Mice were sacrificed after reaching 1000 mm^3^ tumor volume.

To evaluate the effect of [^225^Ac]AJ210 on cell viability, KPC cells were incubated with various dosages of [^225^Ac]AJ210 for 72 h, followed by a viability assessment. The results showed that [^225^Ac]AJ210 induced dose-dependent cell killing, with an IC_50_ of 0.32 µCi/mL (**Figure 7A**). Next, we investigated whether targeting EphA2 in tumors would control tumor growth in a preclinical model of PDAC. Mice with established subcutaneous KPC tumors, which replicate the treatment-resistant nature of human PDAC 32, received a total dose of 1 µCi of [^225^Ac]AJ210 and were monitored for tumor growth. Tumor growth curves demonstrated significantly improved tumor response (**Figure 7B**, p<0.001) and survival with [^225^Ac]AJ210 treatment compared to saline controls (**Figure 7C**, p = 0.0029). Differences in tumor growth became evident as early as 7 days and persisted throughout the study period. H&E staining of lung, liver and kidney sections revealed no visible histological abnormalities in [^225^Ac]AJ210 treated animals compared to the saline treated controls, suggesting minimal acute renal toxicity (**Figure S13-S15**). However, we acknowledge that some weight loss was observed in treated animals (**Figure S16**), and while the underlying cause remains unclear, the possibility of treatment-related toxicity cannot be ruled out. Next, hematological parameters were evaluated to assess systemic toxicity. Mild changes were observed at 3 and 7 days, though these deviations were significant in some parameters at 28 days, suggesting a potential late-stage systemic effect of [^225^Ac]AJ210 (**Figure S17**). These proof-of-principle therapy studies highlight the potential of targeting EphA2 for molecular radiotherapy in PDAC.

## Discussion

In this study, by integrating large-scale genomic analyses from CCLE and TCGA with immunohistochemical validation using PDAC tissue microarrays, we confirm that EphA2 is highly expressed in PDAC, establishing its suitability as a radiotheranostic target. To capitalize on this expression for non-invasive detection and targeted therapy, we developed EphA2-specific binders amenable to radiolabeling with both imaging and radiotherapeutic isotopes. Our results demonstrate that gallium-68-labeled radiotracers bind to EphA2 with high affinity in vitro across all tested PDAC cell lines and enable expression-dependent tumor detection in vivo within 60 min of radiotracer injection. Additionally, we extend beyond imaging by developing and evaluating the first EphA2-targeted alpha-particle therapy using [^225^Ac]-labeled peptides. Given the dense stromal barriers and resistance to conventional treatments seen in PDAC, alpha-particle therapy is particularly promising due to its high LET and short path length, which enable highly localized cytotoxicity while minimizing off-target damage. Our findings demonstrate potent dose-dependent cytotoxicity in PDAC cells and significant tumor growth inhibition in vivo, supporting EphA2-targeted alpha therapy as a novel and effective strategy. To our knowledge, this study is the first to comprehensively establish EphA2 as a radiotheranostic target in PDAC by integrating genomic validation, targeted imaging, and therapeutic efficacy.

Radiological assessment remains the gold standard for PDAC detection, and the lack of efficient diagnostic methods contribute to poor survival rates 1. Accurate diagnosis is crucial for determining surgical candidacy and planning treatment for PDAC. Advances in computed tomography (CT) and magnetic resonance imaging (MRI) techniques, along with increased utilization of cross-sectional imaging, have led to improved diagnosis of pancreatic neoplasms 33. However, [^18^F]-fluorodeoxyglucose (FDG) PET/CT, while useful for identifying local progression, metastatic spread, and evaluating treatment response, suffers from low sensitivity 34, 35. Thus, there is significant interest in developing novel imaging agents for PDAC. In this regard, several targets including MUC5AC, MUC16, CA19.9 and alpha(v)beta(6) targeted antibodies and peptides have been pursued and showed promising results 36-39. Previous efforts to target EphA2, have focused on developing antibody constructs as imaging agents. For instance, [^64^Cu]DOTA-1C1 mAb, which binds both human and mouse orthologs, was used for noninvasive PET imaging of EphA2 levels in various tumor types, including colorectal cancer, melanoma, glioblastoma, and ovarian cancer 19. Similarly, radiolabeled analogs of other anti-EphA2 antibodies, such as DS-8895a and 4B3, have been used to detect EphA2 expression non-invasively in tumors 20, 21. Despite their potential, these antibody-based agents face limitations such as suboptimal pharmacokinetics and higher production costs. To overcome these challenges, low molecular weight analogs have been pursued due to their favorable pharmacokinetics. Peptides like [^18^F]AFP-SWL, [^99m^Tc]-HYNIC-SWL and [^68^Ga]DOTA-SD01 24 have shown specificity for detecting EphA2 expression in melanoma, non-small cell lung and breast cancers but suffered from degradation in plasma and low tumor uptake limiting their application 22, 23. However, none of these agents focused on PDAC.

Recent reports have highlighted high-affinity bicyclic peptides that are stable *in vivo* 17. Derivatives of these bicyclic peptides [^18^F]AlF-ETN and [^68^Ga]Ga-BCY18469 exhibited good image contrast and EphA2 detection in prostate and fibrosarcoma cancer models 25, 26, providing suitable constructs for further development and evaluation. [^68^Ga]AJ201 demonstrates several key advantages over previously studied EphA2-targeting radiotracers, including higher tumor uptake and improved tumor selectivity. PET imaging and biodistribution studies indicate that [^68^Ga]AJ201 exhibits a tumor accumulation profile comparable to [^68^Ga]BCY18469, with slight differences attributed to variations in EphA2 expression across models. However, compared to [^18^F]AlF-ETN, [^68^Ga]Ga-ETN, and [^68^Ga]Ga-BCY6164, [^68^Ga]AJ201 shows significantly higher tumor uptake (SUV 1.31 ± 0.11 vs. 0.9, 0.4, and 0.2, respectively) and lower off-target accumulation in the liver and spleen, supporting its potential as a superior imaging agent. Additionally, [^68^Ga]AJ201 successfully detected small orthotopic tumors (<10 mm^3^), underscoring its potential for early PDAC diagnosis.

Building on the imaging capabilities of these radiotracers, we further developed an alpha-particle-emitting therapeutic analog, [^225^Ac]AJ210. Alpha-particle therapy offers several advantages over beta-emitting radionuclides, particularly for PDAC, which is characterized by a dense stromal microenvironment that limits drug penetration. Unlike beta particles, alpha particles have a short path length (~100 µm) and high linear energy transfer (LET), enabling highly localized tumor cell destruction while sparing surrounding healthy tissues. These properties make alpha therapy particularly well-suited for targeting PDAC, which is resistant to conventional treatments. This study represents a proof-of-principle investigation demonstrating the feasibility of EphA2-targeted alpha-particle therapy. Our results demonstrate that [^225^Ac]AJ210 exerts potent, dose-dependent cytotoxic effects on PDAC cells and significantly inhibits tumor growth in vivo. The therapeutic effects of developed [^225^Ac]AJ210 are likely mediated through three key mechanisms. First, [^225^Ac]AJ210 selectively binds to EphA2-expressing pancreatic tumors, delivering alpha-particle radiation with high LET. Second, EphA2 receptor engagement promotes internalization and degradation, potentially impairing oncogenic signaling and reducing tumor cell invasiveness 40. Further studies are needed to determine whether [^225^Ac]AJ210 modulates EphA2 receptor turnover. Third, EphA2 is known to be expressed on tumor-associated vasculature, where it plays a role in angiogenesis and vascular remodeling 41. By targeting EphA2 on endothelial cells, [^225^Ac]AJ210 could disrupt tumor vasculature, impairing blood supply to the tumor. Additionally, direct alpha-particle irradiation of tumor endothelium could further compromise vascular integrity, contributing to tumor control. Together, these mechanisms highlight [^225^Ac]AJ210 as a dual-action therapeutic agent with both direct tumoricidal and anti-angiogenic properties.

While our results support the potential of [^225^Ac]AJ210 as an effective targeted alpha therapy, kidney retention remains a common concern for peptide-based radiopharmaceuticals due to renal clearance pathways. Our studies revealed elevated kidney uptake of [^68^Ga]AJ201 and [^68^Ga]AJ210, likely due to their hydrophilic residues, a behavior observed in other bicyclic peptides such as BCY18469 and ETN. To address this, we introduced a cleavable amino acid linker designed for enzymatic processing by brush border membrane enzymes in the kidneys 42. However, no significant reduction in kidney uptake was observed within the tested 60-120 min timeframe, suggesting that additional modifications are needed. The cyclic nature of the peptide may hinder linker accessibility, or alternative renal clearance mechanisms may contribute to prolonged retention. Given that previous reports have only demonstrated effective enzymatic cleavage in linear peptides 43-45, future studies will focus on refining linker design and exploring alternative strategies for reducing renal accumulation.

Although preliminary research suggests that α-emitting RPT has a lower impact on kidney function compared to β-emitting RPT 12, further studies are required to assess long-term renal toxicity. Established nephroprotective strategies, such as administering D-lysine, could help mitigate non-specific renal uptake and absorbed dose 46-48. Given that radiotracer uptake does not always directly correlate with toxicity, future studies will incorporate renal function markers and long-term toxicity analyses to better elucidate the implications of renal retention. Systemic exposure to alpha-emitters also poses risks of off-target toxicity in normal organs particularly in the liver, spleen, and bone marrow, necessitating careful dose optimization and monitoring 49, 50. Regarding the observed weight loss, since it was not present in control animals, tumor burden alone is unlikely to be the primary cause. Instead, this may reflect a combination of systemic effects from radiation therapy, mild treatment-related toxicity, or metabolic stress induced by alpha-particle irradiation. Additional investigations, including metabolic and inflammatory markers, will help clarify these effects in future studies. While the therapeutic effects observed here are encouraging, further efforts are required to refine the pharmacokinetic profile of [^225^Ac]AJ210 and optimize its therapeutic window. This includes structural modifications to improve tumor-to-background ratios, dose adjustments to enhance efficacy while minimizing systemic toxicity, and combination strategies to maximize treatment responses.

## Conclusions

In summary, this study establishes EphA2 as a promising radiotheranostic target in PDAC and demonstrates the effectiveness of LMW agents for both imaging and targeted alpha therapy. These agents exhibit high affinity and specificity, leading to significant tumor accumulation and regression with mild toxicity. Our findings provide a strong foundation for EphA2-targeted radiotheranostics, warranting further optimization to enhance therapeutic efficacy and clinical translation.

## Supplementary Material

Supplementary figures and tables.

## Figures and Tables

**Table 1:** Kinetics and distribution of [^68^Ga]AJ201 in mice bearing Panc1 tumor xenografts; data is presented as mean ± SEM (n = 4 or 5) (%ID/g for organs and dimensionless for ratios).

Tissues	5 min	30 min	60 min	60 min with Blocking	90 min	120 min
Blood	8.20 ± 0.92	1.61 ± 0.18	0.49 ± 0.07	0.65 ± 0.11	1.18 ± 0.27	0.90 ± 0.29
Muscle	1.79 ± 0.24	1.11 ± 0.34	0.21 ± 0.05	0.26 ± 0.03	0.37 ± 0.11	0.26 ± 0.04
Tumor	7.06 ± 0.48	6.96 ± 0.31	4.68 ± 0.25	1.08 ± 0.24	6.04 ± 1.46	6.46 ± 0.39
Thymus	4.61 ± 0.51	2.35 ± 0.66	NA	2.28 ± 0.53	1.64 ± 0.42	1.21 ± 0.52
Heart	4.19 ± 1.06	0.97 ± 0.15	0.29 ± 0.08	0.31 ± 0.05	0.76 ± 0.13	0.68 ± 0.17
Lung	8.24 ± 0.93	2.29 ± 0.26	0.65 ± 0.04	0.80 ± 0.14	1.40 ± 0.27	1.15 ± 0.22
Liver	4.25 ± 0.99	1.15 ± 0.19	0.32 ± 0.02	0.48 ± 0.07	0.90 ± 0.17	0.84 ± 0.13
Pancreas	3.24 ± 0.08	0.74 ± 0.10	0.16 ± 0.01	0.30 ± 0.03	0.57 ± 0.15	0.36 ± 0.07
Stomach with contents	1.80 ± 0.39	0.77 ± 0.31	0.15 ± 0.03	0.19 ± 0.06	0.27 ± 0.06	0.30 ± 0.01
Small intestine	3.25 ± 0.30	1.16 ± 0.18	0.49 ± 0.07	0.51 ± 0.08	0.60 ± 0.15	0.45 ± 0.24
Large Intestine	3.67 ± 0.66	1.95 ± 0.98	0.15 ± 0.01	0.93 ± 0.40	1.46 ± 0.35	1.02 ± 0.30
Spleen	7.95 ± 1.66	3.87 ± 1.10	0.74 ± 0.17	0.70 ± 0.11	1.81 ± 0.78	1.55 ± 0.47
Adrenals	4.91 ± 1.83	2.44 ± 0.46	NA	2.36 ± 1.09	1.85 ± 0.46	2.69 ± 0.27
Kidney	37.50 ± 5.93	56.93 ± 1.26	45.45 ± 2.90	71.27 ± 14.16	61.75 ± 11.47	51.98 ± 7.46
Bladder	28.09 ± 12.12	53.77 ± 30.79	48.64 ± 15.54	14.41 ± 13.23	18.27 ± 6.35	19.03 ± 8.08
Femur	2.77 ± 0.13	1.36 ± 0.37	0.28 ± 0.06	0.38 ± 0.05	0.68 ± 0.11	0.83 ± 0.33
Brain	0.40 ± 0.08	0.09 ± 0.01	0.04 ± 0.01	0.04 ± 0.001	0.05 ± 0.01	0.05 ± 0.01
Tumor/blood	0.89 ± 0.14	3.31 ± 1.17	9.86 ± 1.10	1.66 ± 0.37	5.43 ± 1.32	8.36 ± 1.85
Tumor/Muscle	4.12 ± 0.66	6.40 ± 3.6	25.78 ± 6.71	4.40 ± 1.19	16.96 ± 2.11	24.94 ± 1.93
Tumor/Pancreas	2.18 ± 0.14	7.08 ± 2.57	28.78 ± 0.97	3.50 ± 0.70	10.66 ± 0.20	18.74 ± 2.39

## Abbreviations

PDAC: Pancreatic ductal adenocarcinoma
CCLE: Cancer Cell Line Encyclopedia
TCGA: The Cancer Genome Atlas
TMA: Tissue microarrays
EphA2: Ephrin receptor A2
SSTR2: Somatostatin receptor 2, FAP, Fibroblast activation protein
PET: Positron emission tomography
CT: computed tomography
MRI: Magnetic resonance imaging
IHC: Immunohistochemistry, 68Ga, Gallium-68
225Ac: Actenium-225
177Lu: Lutetium-177
mRNA: messenger Ribonucleic acid, PAAD, pancreatic cancer
BLCA: Bladder cancer
READ: Rectal cancer
LUSC: Lung squamous cell carcinoma
THCA: Thyroid cancer
KIRP: Kidney papillary cell carcinoma
LUAD: Lung adenocarcinoma
KIRC: Kidney clear cell carcinoma
CHOL: Bile duct cancer
SKCM: melanoma
LIHC: Liver cancer
KICH: Kidney chromophobe
BRCA: Breast cancer
PRAD: Prostate cancer
DLBC: Large-B-cell lymphoma
LAML: Acute myeloid leukemia
DOTA: 2,2',2'',2'''-(1,4,7,10-tetraazacyclododecane-1,4,7,10-tetrayl)tetraacetic acid
NOTA: 1,4,7-Triazacyclononane-1,4,7-triacetic acid
FDA: Food and drug administration
ROI: Region of Interest
NSG: NOD scid gamma
SEM: standard error of mean
TIAC: Time-integrated activity coefficient
FDG: [18F]-fluorodeoxyglucose
GEP-NET: Gastroenteropancreatic neuroendocrine tumors
PRRT: Peptide receptor radionuclide therapy
LMW: Low molecular weight
NaOAc: sodium acetate
RP-HPLC: Reverse phase high performance liquid chromatography
mCi: millicurie
MALDI-TOF-MS: Matrix assisted laser desorption ionization time of flight mass spectrometry
EtOH: ethanol
ATCC: American type culture collection
cDNA: Complementary DNA
