# Supplementary material for: EphA2-targeted alpha-particle theranostics for enhancing PDAC treatment
Source: Theranostics. 2025 Mar 18;15(10):4229–46. doi: 10.7150/thno.106948 (PMC11984392; doi:10.7150/thno.106948)
Supplement: Supplementary file 1 — Supplementary figures and tables. [file thnov15p4229s1.pdf]

Supplementary Information
For
EphA2-targeted alpha particle theranostics for enhancing PDAC treatment

Ajay Kumar Sharma[1±], *Kuldeep Gupta*[1±], *Akhilesh Mishra*[1], *Gabriela Lofland*[1], *Sophia Y. Chen*[2], *Ian Marsh*[1], *Peyton T Fair*[2], *Robert F. Hobbs*[1], *Todd M. Armstrong*[2], *Elizabeth M. Jaffee*[2], *Edward Gabrielson*[3], *Lei Zheng*[2], *and Sridhar Nimmagadda*[1, 2, 4, 5, *]

Author affiliations:
[1]The Russell H. Morgan Department of Radiology and Radiological Science
[2]Department of Oncology, The Sidney Kimmel Comprehensive Cancer Center and the Bloomberg–Kimmel Institute for Cancer Immunotherapy
[3]Department of Pathology and Oncology
[4]Department of Pharmacology and Molecular Sciences
[5]Division of Clinical Pharmacology, Department of Medicine
Johns Hopkins University School of Medicine,
Baltimore, MD, 21287, USA.
[±]authors contributed equally

*Correspondence to: Sridhar Nimmagadda, Ph.D.
Johns Hopkins Medical Institutions
1550 Orleans Street, CRB II, #492
Baltimore, MD 21287
Phone: 410-502-6244
Fax: 410-614-3147
Email: snimmag1@jhmi.edu

Keywords: Pancreatic cancer, PET, Imaging, Radiotherapy, Peptide radiopharmaceuticals, Gallium-68, Alpha-particle therapy

A **B**

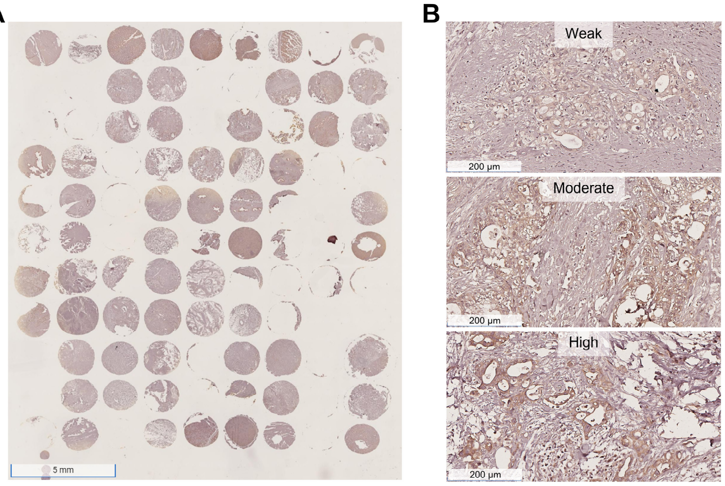

C

EphA2 intensity	Scores	Cytoplasm, N (%)	membrane, N (%)
Weak	1	8 (29 %)	7 (27 %)
Moderate	2	7 (25 %)	9 (35 %)
High	3	12 (43 %)	9 (35 %)
Negative	0	1 (4%)	1 (4%)
Ignored	Not scored	38	38
Missing	Not scored	30	30
No panc	Not scored	3	3
NA	Not scored	0	2

Ignored, not able to score
Missing, tissue spot was missing from TMA slide
No Panc, tissue spot was not for Pancreatic cancer
NA, not available for scoring

Figure S1. Immunohistochemistry analysis of EphA2 expression in a Tissue Microarray (TMA) of PDACs. A) A full TMA slide showcasing IHC staining for EphA2 across multiple PDAC samples demonstrating the variability in EphA2 expression among the tumor samples. **B)** Representative images from the TMA slide displaying tumor sections with varying levels of EphA2 expression highlighting the differences in staining intensity, ranging from low to high expression of EphA2. **C)** Quantitative analysis of the PDAC samples based on IHC staining intensity for membranous EphA2 expression. A scoring system was employed to categorize the samples, revealing that 95% of the PDAC samples exhibited positive staining for membranous EphA2. The scoring of EphA2 was determined by the intensity and distribution of staining in tumor cells. A score of 0 indicates no staining or weak staining in less than 10% of tumor cells, while a score of 1 reflects weak to moderate membranous or membranous and cytoplasmic staining in more than 10% of tumor cells. A score of 2 signifies moderate membranous or membranous and cytoplasmic staining in more than 50% of tumor cells, and a score of 3 represents strong membranous or membranous and cytoplasmic staining in more than 80% of tumor cells.

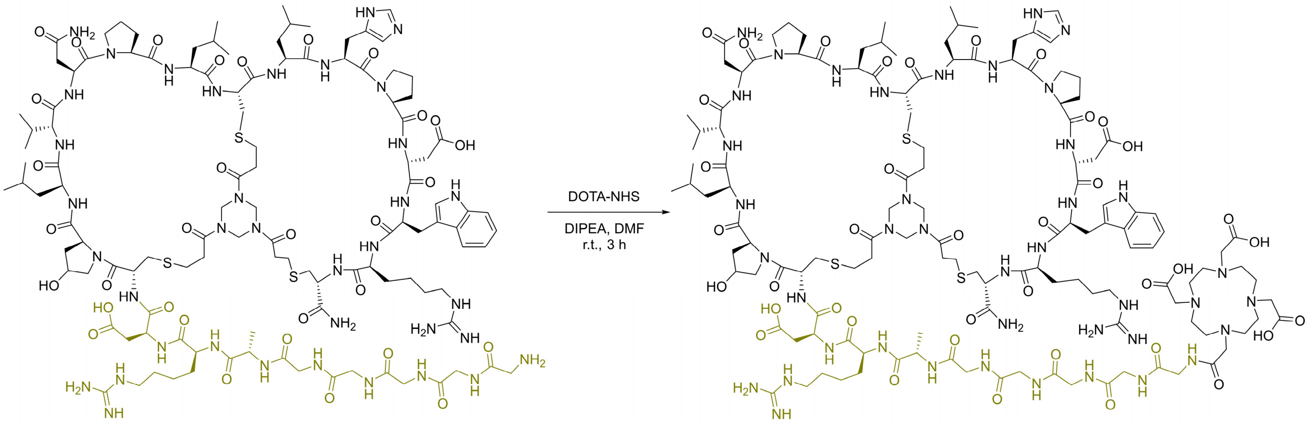

AJ200 AJ210

Scheme S1. Synthesis of AJ210 using a DOTA Chelator. The DOTA chelator was conjugated to AJ200 through a reaction mediated by N,N-Diisopropylethylamine (DIPEA), resulting in the formation of AJ210. The synthesized AJ210, featuring the DOTA chelator, was designed to facilitate the radiolabeling with Ga-68 and Ac-225, for imaging and therapeutic applications, respectively. In the structures, black color represents binding moiety and parakeet color reparents the linker.

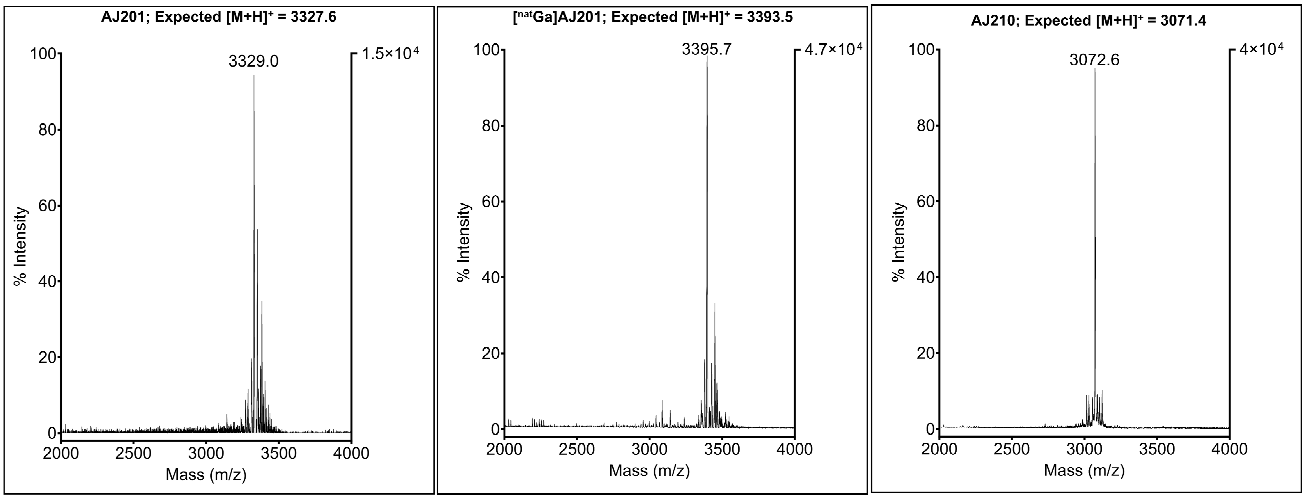

Figure S2. Characterization of AJ201, [natGa]AJ210 and AJ210 peptides using MALDI-TOF Mass spectrometry.

Table S1. Parameters for SPR study

Ligand (L)	FC	Analyte (A)	Ligand Binding	MW_L (Da)	MW_A (Da)	Stochiometric Ratio	R_{max}
Human EphA2	2	AJ201	750	56900	3328.8	1:1	43.9
Mouse EphA2	4	AJ201	750	58000	3328.8	1:1	43.9
Human EphA2	2	AJ210	850	56900	3072.5	1:1	40.1

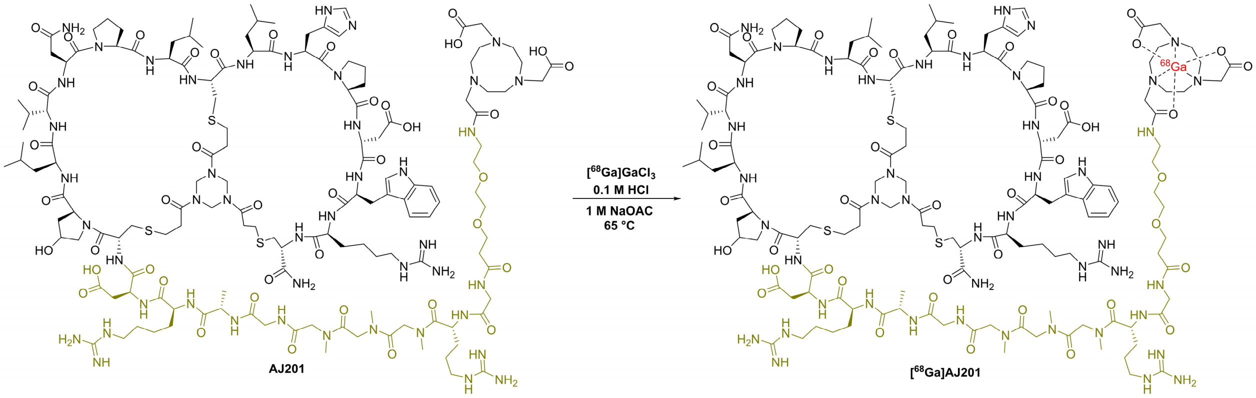

Scheme S2. Synthesis scheme of [^{68}Ga]AJ201. The labeling of AJ201 with ^{68}Ga was conducted with heating at 65 °C for 10 min. This ensured efficient incorporation of the radioactive isotope. For comparison, the labeling process using natural Gallium (^{nat}Ga) was performed under similar conditions but extended to 30 min at 65 °C. This step verifies the robustness of the labeling procedure and provides a reference for the efficiency of radiolabeling with ^{68}Ga. In the structures, black color represents binding moiety, parakeet color reparents the linker and red color represents the radiometal.

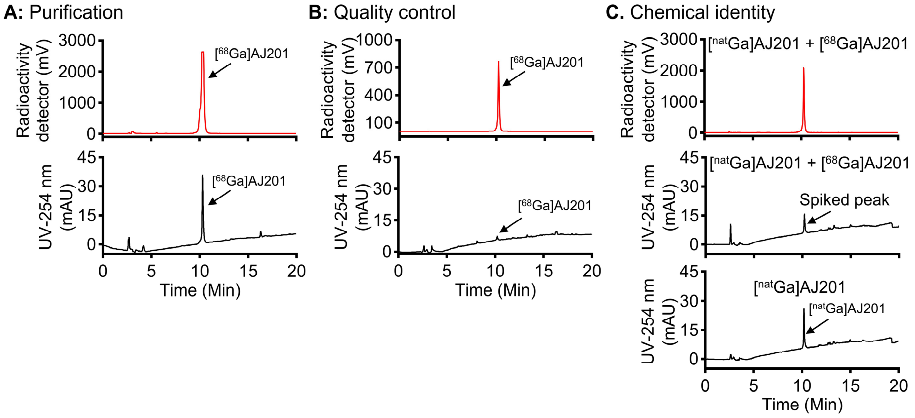

Figure S3. Radiolabeling and characterization of [^{68}Ga]AJ201. A) HPLC chromatograms for the purification process of [^{68}Ga]AJ201, showing the separation of the radiolabeled product from small impurities and unreacted ^{68}Ga **B)** HPLC chromatogram of the purified [^{68}Ga]AJ201 after formulation, confirming the >99% radiochemical purity of the radiolabeled product. **C)** HPLC chromatograms confirming the chemical identity of [^{68}Ga]AJ201 by comparing it with [^{nat}Ga]AJ201. The matching retention times and peak profiles between [^{68}Ga]AJ201 and [^{nat}Ga]AJ201 confirm the successful and specific incorporation of ^{68}Ga into the AJ201 peptide, validating the radiolabeling process.

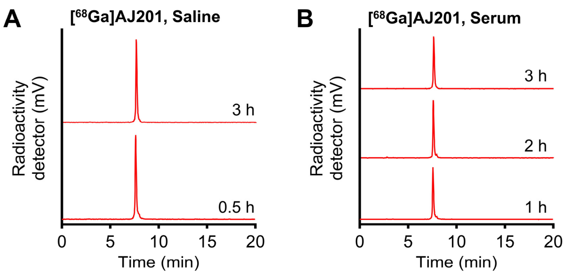

Figure S4. Stability study of [^{68}Ga]AJ201. A) HPLC chromatograms demonstrating the stability of [^{68}Ga]AJ201 in saline at 0.5 and 3 h at 37 ˚C, shows single peak and consistent retention time, indicating that [^{68}Ga]AJ201 remains stable without noticeable degradation or impurity formation. **B)** HPLC chromatograms of [^{68}Ga]AJ201 after the incubation in human serum at 1, 2 and 3 h at 37 ˚C, shows single peak and consistent retention time, indicating that [^{68}Ga]AJ201 remains stable without noticeable degradation or impurity formation.

Table S2. Biodistribution of [^{68}Ga]AJ201 in mice bearing pancreatic tumor xenografts at 60 min post-injection; data is presented as mean ± SEM (n = 4 or 5) of %ID/g.

Tissues	CFPAC	Panc1005	BxPC3	Hs766T	AsPC1	Su8686
**Blood**	0.69 ± 0.21	0.44 ± 0.05	0.58 ± 0.04	1.39 ± 0.30	3.32 ± 0.24	0.71 ± 0.18
**Muscle**	0.21 ± 0.04	0.11 ± 0.01	0.18 ± 0.01	0.46 ± 0.13	0.41 ± 0.03	0.16 ± 0.04
**Tumor**	2.4 ± 0.21	5.31 ± 0.90	2.72 ± 0.37	7.97 ± 2.83	4.01 ± 0.46	7.66 ± 1.00
**Heart**	0.34 ± 0.08	0.20 ± 0.02	0.29 ± 0.01	0.90 ± 0.25	1.25 ± 0.10	0.25 ± 0.06
**Lung**	0.89 ± 0.20	0.54 ± 0.05	0.70 ± 0.04	1.71 ± 0.55	2.78 ± 0.13	0.76 ± 0.16
**Liver**	0.43 ± 0.09	0.29 ± 0.03	0.42 ± 0.02	0.89 ± 0.21	1.59 ± 0.07	0.34 ± 0.06
**Pancreas**	0.32 ± 0.07	0.21 ± 0.03	0.25 ± 0.02	0.61 ± 0.22	0.65 ± 0.04	0.22 ± 0.05
**Small intestine**	0.51 ± 0.10	0.25 ± 0.03	0.32 ± 0.02	0.62 ± 0.02	1.12 ± 0.08	0.29 ± 0.05
**Spleen**	0.87 ± 0.15	0.52 ± 0.05	0.64 ± 0.05	2.75 ± 0.65	1.61 ± 0.11	0.60 ± 0.16
**Kidney**	69.08 ± 4.56	67.05 ± 6.90	59.03 ± 7.17	52.63 ± 4.07	59.57 ± 2.86	37.69 ± 2.64
**Tumor/blood**	4.53 ± 0.90	12.93 ± 2.38	4.86 ± 0.92	5.47 ± 0.81	1.20 ± 0.06	14.2 ± 3.89
**Tumor/Muscle**	12.1 ± 1.45	50.09 ± 10.21	15.24 ± 2.14	17.17 ± 2.33	9.95 ± 1.49	57.99 ± 11.48
**Tumor/Pancreas**	8.68 ± 1.24	26.90 ± 5.62	11.01 ± 1.71	13.76 ± 2.30	6.23 ± 0.65	39.77 ± 7.21

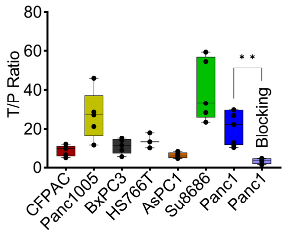

Figure S5. *Ex vivo* biodistribution study at 60 min post-injection, highlighting the tumor-to-pancreas ratio of [^{68}Ga]AJ201 in various pancreatic tumor xenograft models at 60 min post-injection. This provides insight into the specificity and uptake of [^{68}Ga]AJ201 in tumor tissues compared to normal pancreatic tissue across different models. data is shown as box and whisker plots (median ± IQR) showing all data points (n = 4-5). **, P<0.01 by unpaired student's t-test.

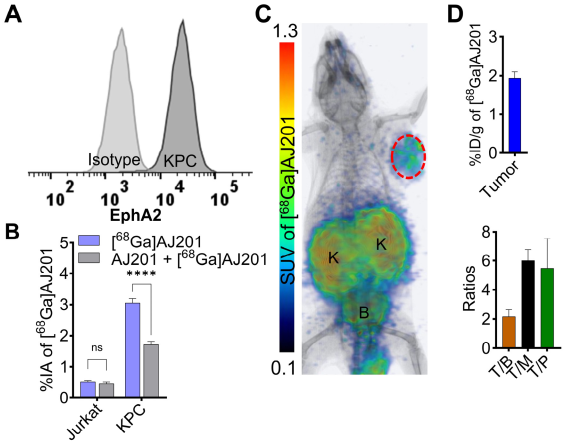

Figure S6. ***In vitro*** **and** ***in vivo*** **evaluation of [^{68}Ga]AJ201 in KPC syngeneic tumor model. A)** Validation of EphA2 expression in KPC cells by flow cytometry. **B)** *In vitro* tracer uptake assay of [^{68}Ga]AJ201 in KPC cells, with and without peptide blocking, demonstrating the specific binding of [^{68}Ga]AJ201 to EphA2, as indicated by reduced tracer uptake in the presence of an excess of unlabeled peptide. **C)** Whole-body static PET imaging of a KPC syngeneic tumor-bearing mouse at 60 min post-injection of the radiotracer [^{68}Ga]AJ201, showing the accumulation of the radiotracer in the tumor area. **D)** Ex vivo biodistribution study of [^{68}Ga]AJ201 at 60 min post-injection in the KPC syngeneic tumor model, providing a quantitative analysis of radiotracer accumulation in tumor (top), demonstrating the preferential uptake of [^{68}Ga]AJ201 in the tumor compared to other organs (bottom). T/B is tumor-to-blood; T/M is tumor-to-muscle and T/P is tumor-to-pancreas ratios.

Table S3. Biodistribution of [^{68}Ga]AJ201 in KPC syngeneic tumor at 60 min post-injection; data is presented as mean ± SEM (n = 5) of %ID/g.

Tissues	KPC
**Blood**	0.75 ± 0.15
**Muscle**	0.28 ± 0.08
**Tumor**	1.51 ± 0.28
**Heart**	0.30 ± 0.06
**Lung**	0.78 ± 0.13
**Liver**	2.46 ± 0.77
**Pancreas**	0.22 ± 0.04
**Stomach**	0.95 ± 0.26
**Small intestine**	0.62 ± 0.25
**Large Intestine**	0.61 ± 0.28
**Spleen**	0.71 ± 0.30
**Kidney**	50.29 ± 9.75
**Bladder**	74.89 ± 15.33
**Femur**	0.71 ± 0.22
**Brain**	0.04 ± 0.01
**Tumor/blood**	2.17 ± 0.46
**Tumor/Muscle**	6.00 ± 0.77
**Tumor/Pancreas**	6.67 ± 2.18

Table S4. Predicted time-integrated activity coefficients (TIACs) and absorbed dose coefficients of [^{68}Ga]AJ201 in human organs

Tissues	Residence time (h)	Organ dose (rem/mCi)
**Blood**	0.05152	n/a
**Muscle**	0.07064	0.00962
**Tumor**	n/a	n/a
**Thymus**	0.0002384	0.020165
**Heart**	0.001764	0.032745
**Lung**	0.01099	0.04292
**Liver**	0.01723	0.023976
**Pancreas**	0.0006321	0.027084
**Stomach with contents**	0.0004823	0.015799
**Small intestine**	0.003647	0.016391
**Large Intestine**	0.001341	0.018389
**Spleen**	0.002451	0.036445
**Adrenals**	0.0002761	0.06253
**Kidney**	0.08395	0.3885
**Bladder**	0.006394	0.15133
**Femur**	0.005607	0.014208
**Brain**	0.0008446	0.0032153

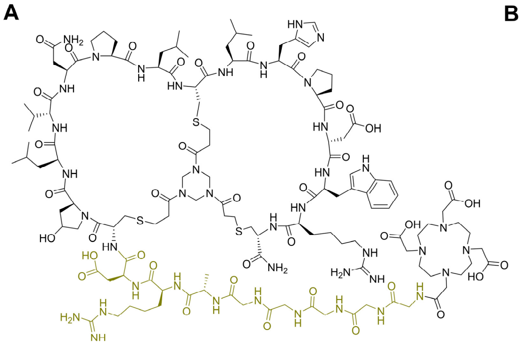

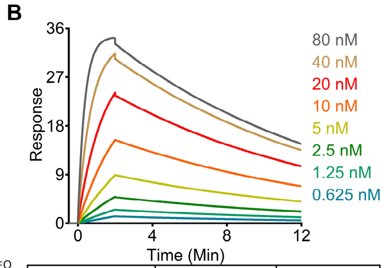

K_{on} (1/Ms) x10^5	K_{off} (1/s) x10^{-3}	$K_D = K_{off}/K_{on}$ (nM)
5.607	1.364	2.4

Figure S7. Structure and *in vitro* characterization of AJ210. A) Structure of the bicyclic peptide AJ210, which contains the DOTA as a bifunctional chelator as a for radiolabeling with ^{68}Ga and ^{225}Ac. In the structure, black color represents binding moiety and parakeet color reparents the linker. **B)** Surface Plasmon Resonance (SPR) analysis demonstrating the binding affinity of AJ210 for EphA2 using recombinant human EphA2 protein. The SPR sensorgrams show the interaction kinetics between AJ210 and EphA2, providing data on the association and dissociation rates, which reflect the affinity of AJ210 for the EphA2 receptor.

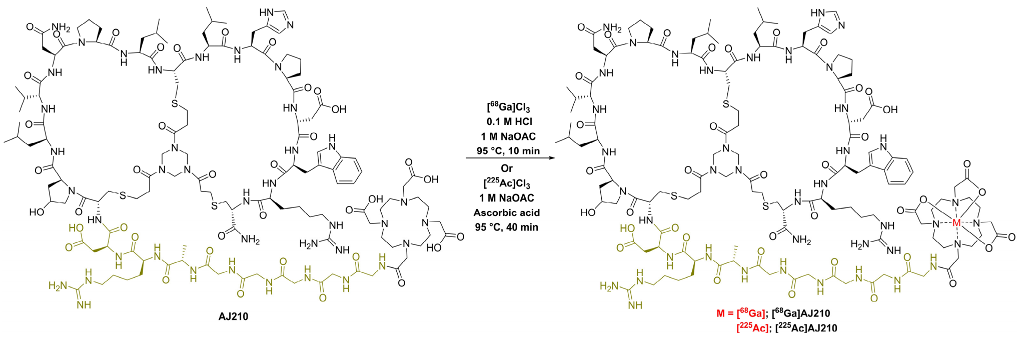

Scheme S3. Synthesis scheme of radiometalation reaction to form [^{68}Ga]AJ210 and [^{225}Ac]AJ210. The synthesis of [^{68}Ga]AJ210 involves radiolabeling AJ210 with ^{68}Ga. The labeling reaction is performed by heating at 95 °C for 10 min, resulting in the formation of [^{68}Ga]AJ210. The synthesis of [^{225}Ac]AJ210 involves radiolabeling AJ210 with ^{225}Ac. This reaction is conducted by heating at 95 °C for 40 min, yielding [^{225}Ac]AJ210. The radiolabeled compounds [^{68}Ga]AJ210 and [^{225}Ac]AJ210 are prepared for different applications. [^{68}Ga]AJ210 is utilized for PET imaging and [^{225}Ac]AJ210 is employed as a radiotherapeutic agent. In the structures, black color represents binding moiety, parakeet color reparents the linker and red color represents the radiometal.

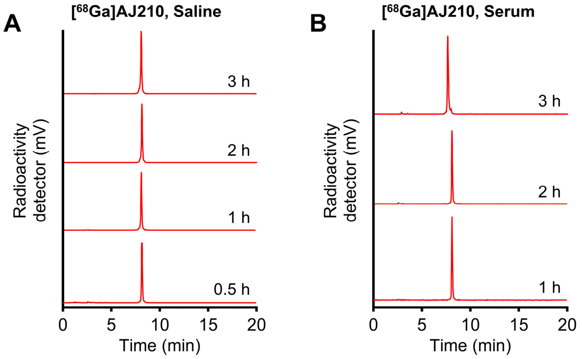

Figure S8. Stability analysis of [^{68}Ga]AJ210. A) HPLC chromatograms of [^{68}Ga]AJ210 in saline at 37 ˚C over 0.5, 1, 2 and 3 h exhibit a single peak with consistent retention time, confirming its stability without detectable degradation or impurity formation. **B)** HPLC chromatograms of [^{68}Ga]AJ210 following incubation in human serum at 37 ˚C for 1, 2 and 3 h, shows a single peak with unchanged retention time, indicating that [^{68}Ga]AJ201 remains stable without significant degradation or impurity formation.

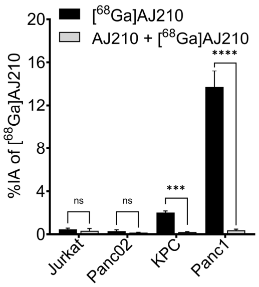

Figure S9. Binding of [^{68}Ga]AJ210 to various human and mouse cell lines expressed as the percent of incubated activity (%IA). Cells were incubated with 1 µCi of [^{68}Ga]AJ210 at 4 °C for 1 h. The data demonstrate the binding efficiency of [^{68}Ga]AJ210 across different cell types. Co-incubation with 2 µM of non-radioactive AJ210 (blocking dose) significantly reduces the radiotracer uptake in EphA2-expressing cells, confirming the specificity of [^{68}Ga]AJ210 for EphA2. Statistics were calculated using two-way ANOVA,; ns, $P \geq 0.05$; ***, $P \leq 0.001$; ****, $P \leq 0.0001$.

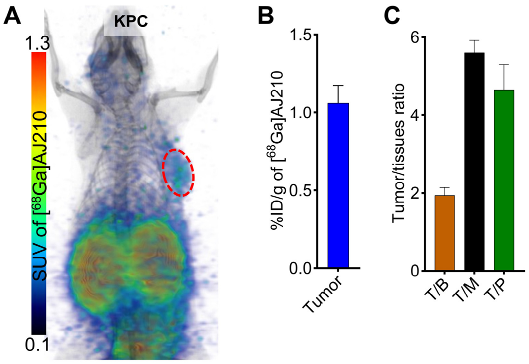

Figure S10. *In vivo* evaluation of [^{68}Ga]AJ210 in KPC syngeneic tumor model. A) Whole-body static PET image obtained 60 min after injection of [^{68}Ga]AJ210 into a KPC syngeneic tumor-bearing mouse. The PET image shows the distribution of the radiotracer throughout the body and providing a visual representation of the tracer's localization in the tumor and other tissues. **B)** Tumor uptake from an ex vivo biodistribution study performed 60 min post-injection of [^{68}Ga]AJ210. **C)** tumor-to-tissues ratios from an ex vivo biodistribution study performed 60 min post-injection of [^{68}Ga]AJ210. T/B is tumor-to-blood; T/M is tumor-to-muscle and T/P is tumor-to-pancreas ratios.

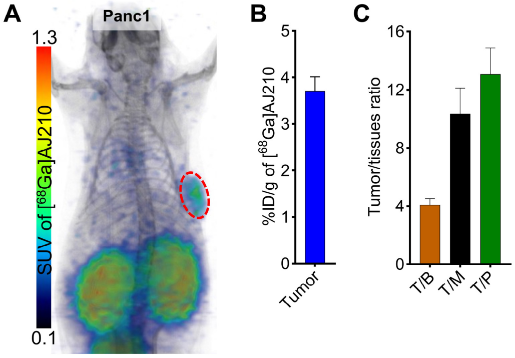

Figure S11. *In vivo* evaluation of [^{68}Ga]AJ210 in Panc1 tumor model. A) Whole-body static PET image obtained 60 min after injection of [^{68}Ga]AJ210 into a Panc1 bearing tumor xenograft model. The PET image shows the distribution of the radiotracer throughout the body and providing a visual representation of the tracer's localization in the tumor and other tissues. **B)** Tumor uptake from an ex vivo biodistribution study performed 60 min post-injection of [^{68}Ga]AJ210. **C)** tumor-to-tissues ratios from an ex vivo biodistribution study performed 60 min post-injection of [^{68}Ga]AJ210. T/B is tumor-to-blood; T/M is tumor-to-muscle and T/P is tumor-to-pancreas ratios.

Table S5. Biodistribution of [^{68}Ga]AJ210 in mice bearing pancreatic tumor xenografts at 60 min post-injection; data is presented as mean ± SD (n = 5) of %ID/g.

Tissues	KPC	Panc1
Blood	0.56 ± 0.14	0.96 ± 0.33
Muscle	0.19 ± 0.04	0.40 ± 0.16
Femur	0.29 ± 0.07	0.38 ± 0.08
Tumor	1.06 ± 0.25	3.72 ± 0.66

Lung	0.57 ± 0.13	0.39 ± 0.08
Heart	0.24 ± 0.05	0.84 ± 0.14
Liver	0.26 ± 0.05	0.34 ± 0.06
Small intestine	0.64 ± 0.47	0.62 ± 0.22
Stomach	0.17 ± 0.07	0.09 ± 0.03
Spleen	0.35 ± 0.07	0.74 ± 0.22
Pancreas	0.24 ± 0.06	0.30 ± 0.08
Kidney	59.9 ± 16.35	24.40 ± 9.98
Tumor/blood	1.94 ± 0.44	4.09 ± 0.97
Tumor/Muscle	5.61 ± 0.69	10.33 ± 4.01
Tumor/Pancreas	4.65 ± 1.46	13.05 ± 4.11

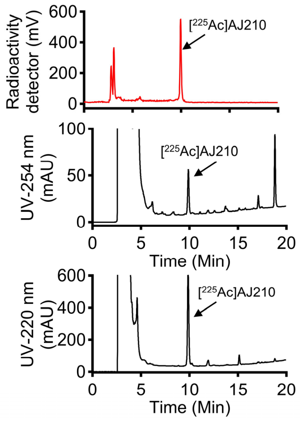

Figure S12. Radiosynthesis of [^{225}Ac]AJ210. HPLC chromatograms for the purification process of [^{225}Ac]AJ210, showing the separation of the radiolabeled product from small impurities and unreacted ^{225}Ac.

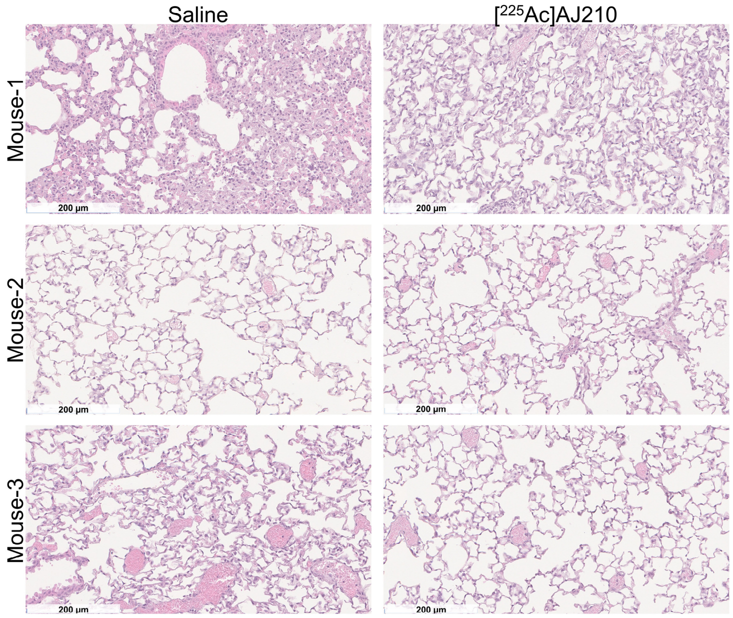

Figure S13. H&E-stained histological sections of lungs from mice treated with saline as a control and [^{225}Ac]AJ210. The slides show normal lung tissue morphology with no significant pathological changes in saline and [^{225}Ac]AJ210 treated animals (n = 3).

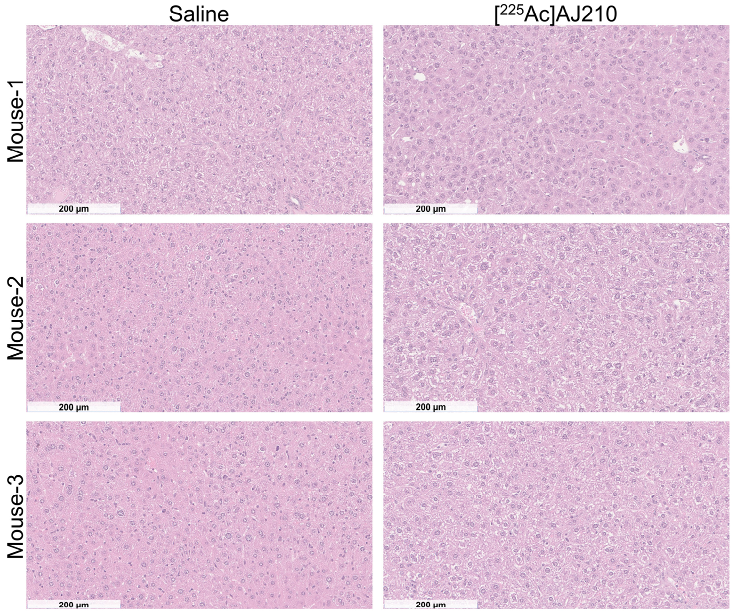

Figure S14. H&E-stained histological sections of liver from mice treated with saline as a control and [^{225}Ac]AJ210. The slides show normal liver tissue morphology with no significant pathological changes in saline and [^{225}Ac]AJ210 treated animals (n = 3).

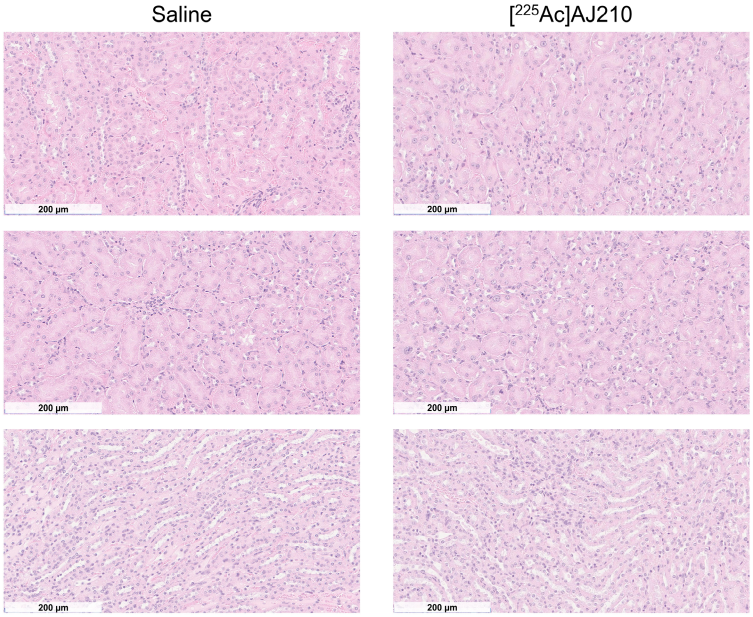

Figure S15. H&E-stained histological sections of kidney from mice treated with saline as a control and [^{225}Ac]AJ210. The slides show normal kidney tissue morphology with no significant pathological changes in saline and [^{225}Ac]AJ210 treated animals (n = 3).

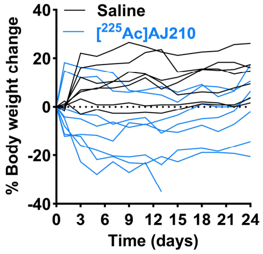

Figure S16. Percentage body weight change during the radiotherapeutic evaluation of [^{225}Ac]AJ210 in KPC bearing mice; The figure displays the percentage change in body weight for individual KPC-bearing mice during the radiotherapeutic evaluation of [^{225}Ac]AJ210. Each line represents data from a single animal over the course of the study. One mouse experienced more than 20% weight loss and was sacrificed on day 13 due to health concerns.

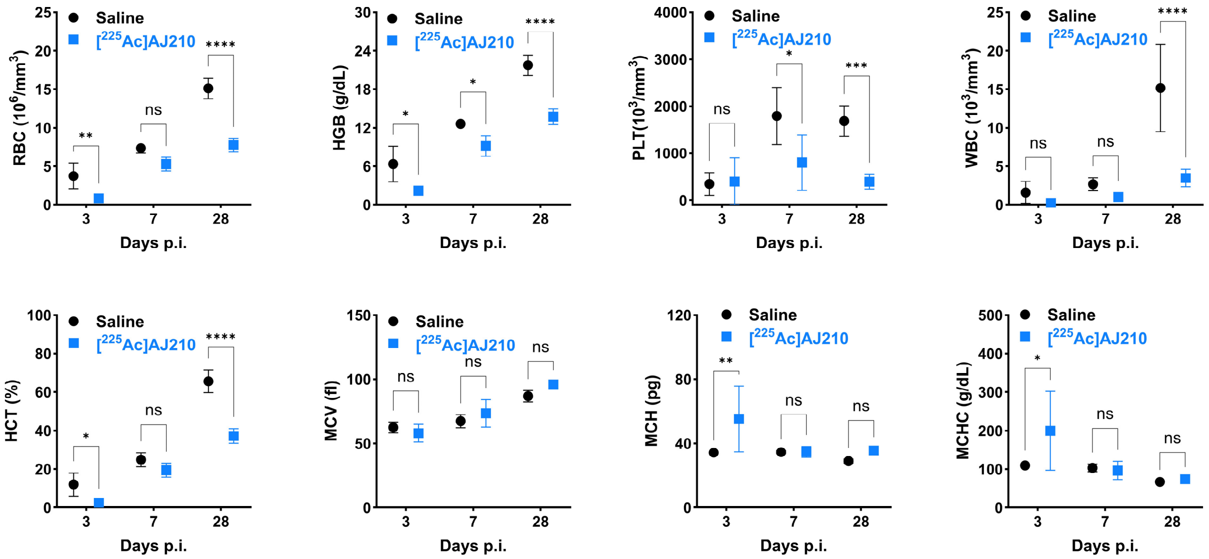

Figure S17. Hematological parameters of mice treated with saline (control) and [^{225}Ac]AJ210. Parameters analyzed include red blood cell count (RBC), hemoglobin concentration (HGB), platelet count (PLT), white blood cell count (WBC), hematocrit (HCT), mean corpuscular volume (MCV), mean corpuscular hemoglobin (MCH), and mean corpuscular hemoglobin concentration (MCHC). Data indicate changes in the parameter, suggesting marginal hematological impact of [^{225}Ac]AJ210 treatment. Data represented as mean ± SD (n = 3 or 4); Statistics were calculated using two-way ANOVA,; ns, $P \geq 0.05$; *, $P \leq 0.05$; **, $P \leq 0.01$; ***, $P \leq 0.001$; ****, $P \leq 0.0001$.
